# Impact of SGLT2 Inhibitors on Mortality Across Different Populations: A Systematic Review and Meta-Analysis

**DOI:** 10.3390/ijms27073168

**Published:** 2026-03-31

**Authors:** Dana Emilia Movila, Alexandru Catalin Motofelea, Simona Ruxanda Dragan, Adalbert Schiller, Adina Ionac, Nadica Motofelea, Florina Caruntu

**Affiliations:** 1 University Clinic of Internal Medicine and Ambulatory Care, Prevention and Cardiovascular Recovery, Department VI-Cardiology, “Victor Babes” University of Medicine and Pharmacy, 300041 Timisoara, Romania; dana.movila@umft.ro (D.E.M.); simona.dragan@umft.ro (S.R.D.); 2 Research Centre of Timisoara Institute of Cardiovascular Diseases, “Victor Babes” University of Medicine and Pharmacy, 300041 Timisoara, Romania; 3 Centre for Molecular Research in Nephrology and Vascular Disease/MOL-NEPHRO-VASC, “Victor Babes” University of Medicine and Pharmacy Timisoara, 300041 Timisoara, Romania; schiller.adalbert@umft.ro; 4 Department of Cardiology, “Victor Babes” University of Medicine and Pharmacy, 300041 Timisoara, Romania; adina.ionac@umft.ro; 5 Department of Obstetrics and Gynecology, “Victor Babes” University of Medicine and Pharmacy, Eftimie Murgu Sq. No. 2, 300041 Timisoara, Romania; nadica.motofelea@umft.ro; 6 First Department of Internal Medicine, Medical Semiology II, “Victor Babes” University of Medicine and Pharmacy, 300041 Timisoara, Romania; caruntu.florina@umft.ro; 7 Multidisciplinary Heart Research Center, “Victor Babes” University of Medicine and Pharmacy, 300041 Timisoara, Romania

**Keywords:** SGLT2 inhibitor, all-cause mortality, cardiovascular mortality, death

## Abstract

Sodium-glucose cotransporter-2 (SGLT2) inhibitors offer glucose-lowering, cardio-protective and reno-protective properties. Mortality rates constitute a central endpoint for understanding the overall clinical value of SGLT2 inhibitors. This systematic review and meta-analysis aims to compare mortality outcomes associated with SGLT2 inhibitors across different populations. A systematic search was performed in four databases—PubMed, Scopus, Web of Science (WOS) and Cochrane CENTRAL—in March 2025. We strictly included randomized controlled trials (RCTs) that compared patients who received SGLT2is to control patients regarding mortality outcomes. All-cause mortality up to one year, all-cause mortality more than one year, cardiovascular mortality, renal mortality and in-hospital mortality were the extracted outcomes. Finally, RevMan (5.4) was adopted for meta-analysis, and OpenMeta analyst software was adopted for meta-regression. Fifty clinical trials met the eligibility criteria of the current systematic review and meta-analysis. SGLT2 inhibitors significantly reduced all-cause mortality in studies with follow-up of up to one year (RR = 0.89, 95% CI [0.80–0.99], *p* = 0.03). This early survival benefit was primarily driven by the subgroup of patients treated during acute cardiac decompensation (RR = 0.76, 95% CI [0.60–0.97], *p* = 0.03). Furthermore, long-term follow-up beyond one year showed a significant reduction in all-cause mortality (RR = 0.89, 95% CI [0.85–0.94], *p* < 0.0001), particularly among patients with chronic heart failure, chronic kidney disease (CKD), and diabetes mellitus (DM) with established cardiovascular disease (CVD) (following sensitivity analyses). Cardiovascular mortality was also significantly reduced overall (RR = 0.88, 95% CI [0.84–0.94], *p* < 0.0001), with the greatest benefit observed in chronic heart failure and CKD subgroups. SGLT2 inhibitors as a class provide a consistent and significant reduction in all-cause mortality across both short-term (up to one year) and long-term follow-up. The early survival benefit is particularly evident when initiated during acute cardiac decompensation, while the long-term benefit extends to chronic heart failure, CKD, and high-risk DM. Future well-designed trials are needed to address the impact of less-explored SGLT2 inhibitors and understudied populations.

## 1. Introduction

Sodium-glucose cotransporter-2 (SGLT2) inhibitors have emerged as a treatment for various medical conditions in the past decade, due to their glucose-lowering, cardio-protective and reno-protective properties [1]. Canagliflozin was the first SGLT2 inhibitor to be approved by the United States Food and Drug Administration (US FDA) for the treatment of type 2 diabetes mellitus (DM) hyperglycemia in 2013 [2]. In addition to canagliflozin, various SGLT2 inhibitors were approved in subsequent years, such as empagliflozin, dapagliflozin and ertugliflozin [3]. In 2021, dapagliflozin was the first SGLT2 inhibitor to be approved by the US FDA for chronic kidney disease (CKD) regardless of diabetes/hyperglycemia status [4]. In 2020, the US FDA approved dapagliflozin for adult patients suffering from heart failure with reduced ejection fraction (HFrEF) regardless of diabetes/hyperglycemia status [5].

The American Diabetes Association (ADA) currently recommends SGLT2 inhibitors as a first choice for patients with heart failure, patients with atherosclerotic cardiovascular disease and those at high risk of cardiovascular diseases [6]. SGLT2 inhibitors are also recommended by the European Society of Cardiology (ESC) and the ACC/AHA/HFSA 2022 Heart Failure Guideline as a class I therapy for HFrEF [7,8,9]. The BMJ CKD SGLT2 inhibitors guidelines strongly recommended the class for adults at high or very-high risk of CKD progression and complications [10]. Data from 2015 and 2020 indicated that 9,900,981 individuals in the United States met the KDIGO guidelines as eligible patients for SGLT2 inhibitors [11]. In 2020, the KDIGO guidelines recommended SGLT2 inhibitors for patients with CKD and type 2 DM; however, the eligibility criteria were expanded in 2024 to include any adult patient with CKD, irrespective of glycemic status [12,13]. SGLT2 inhibitors have been endorsed as a part of the treatment protocol during acute cardiac decompensation [14]. Additionally, the class has been investigated as an add-on therapy in critically ill and COVID-19 patients [15,16,17].

Mortality rates constitute a central endpoint for understanding the overall clinical value of SGLT2 inhibitors as an emerging treatment class. A previously published systematic review and meta-analysis concluded that SGLT2 inhibitor therapy significantly reduced morbidity and mortality among patients with cardiovascular and renal diseases [18]. The growing literature investigating the effects of SGLT2 inhibitors across diverse populations, along with the guidelines expanding the eligibility criteria for SGLT2 inhibitor therapy, has warranted the need for an updated meta-analysis. Accordingly, this study aims to conduct an up-to-date systematic review and double-arm meta-analysis to compare mortality outcomes associated with SGLT2 inhibitors across different populations, with subgroup analyses evaluating how mortality effects differ according to the underlying disease status.

## 2. Methods

### 2.1. Protocol and Registration

The current systematic review and meta-analysis concerning the effect of sodium-glucose cotransporter-2 inhibitors (SGLT2is) on mortality outcomes among different populations was registered with PROSPERO (CRD420261281179). This systematic review was designed and reported in alignment with the Preferred Reporting Items for Systematic Reviews and Meta-Analyses (PRISMA) guidelines [19].

### 2.2. Literature Search

We systemically searched four electronic databases including PubMed, Web of Science, Scopus and Cochrane CENTRAL for research work related to mortality outcomes among populations who have received SGLT2is. Our electronic search was conducted on 12 March 2026, using the following search strategy: (SGLT2 OR SGLT-2 OR “sodium glucose transport” OR “sodium-glucose transport” OR “sodium glucose cotransporter” OR “sodium-glucose cotransporter” OR “sodium glucose co-transporter” OR “sodium-glucose co-transporter” OR Empagliflozin OR Dapagliflozin OR Canagliflozin OR Ertugliflozin OR Sotagliflozin OR Ipragliflozin OR Luseogliflozin OR Tofogliflozin OR Bexagliflozin OR Licogliflozin) AND (Mortality OR mortalities OR death* OR Fatality). The detailed search strategy for each database is outlined in Supplementary Table S1.

### 2.3. Study Selection

EndNote X9, the reference manager software, was adopted to remove duplicates, keeping only one original record. The final set of records was imported to Microsoft Excel, where two authors independently screened titles and abstracts of these records. The full texts of records that were potentially eligible for inclusion were sought for retrieval to be further examined for eligibility. Clinical trials that compared patients who received SGLT2is to control patients regarding mortality outcomes were considered eligible for inclusion. Excluded studies included review articles, observational studies, protocols, studies written in languages other than English, conference abstracts, studies with no available full texts, and studies that did not report mortality as a separate outcome suitable for pooling as risk ratios. In addition, studies in which the entire population initially received SGLT2is and then comparisons were made between adherent and non-adherent patients were excluded. Disagreements were resolved through discussion with a senior author.

### 2.4. Data Extraction

General characteristics of the included studies, baseline characteristics of their population and mortality outcomes were extracted to a Microsoft Excel sheet by two authors independently. General characteristics included last name of the first author, year of publication, trial name, the country/countries where the trial was carried out, the included population of each study and SGLT2i intervention details along with control details and number of included participants in each study group. Baseline characteristics included age, sex, weight, body mass index (BMI), smoking status and the reported comorbidities. All-cause mortality up to one year, all-cause mortality more than one year, cardiovascular mortality, renal mortality and in-hospital mortality were extracted. For temporal stratification, follow-up durations of exactly 12 months (or 52 weeks) and under were categorized as short-term (up to one year). Follow-up durations strictly greater than 12 months were categorized as long-term (more than one year).

### 2.5. Analytical Approach

Double-arm meta-analysis was conducted using RevMan 5.4 software. Random-effect model was the adopted model for all outcomes because different populations were included in the current meta-analysis. Outcomes were pooled as risk ratios and 95% confidence intervals (CIs). Heterogeneity between studies was concluded whenever χ^2^ test showed a *p* value less than 0.1. By removing one study in each scenario, leave-one-out test was adopted to resolve heterogeneity between studies in a specific outcome or within subgroups. Subgroup analyses were conducted according to the specific SGLT2i used in each study and the population receiving the SGLT2i intervention, as defined by the inclusion criteria of the included studies. A *p* value below 0.05 was considered a statistically significant finding [20]. Open Meta-Analyst software [21] was also used to perform meta-regression analyses for the incidence of mortality based on the mean differences in baseline weight and BMI values between study groups. Means and standard deviations of weight and BMI were estimated, whenever applicable, according to Luo et al. (2018) [22] and Wan et al. (2014) [23] for inclusion in the meta-regression models. If the population of a given study exhibited skewness according to Shi et al. (2023) [24] regarding weight or BMI, that study was excluded from the meta-regression model for the corresponding variable. Potential publication bias and small-study effects were evaluated via visual inspection of funnel plots and Egger’s regression test. In accordance with Cochrane methodological guidelines [25,26], these assessments were strictly reserved for meta-analyses including 10 or more studies, as tests for asymmetry are substantially underpowered to distinguish chance from true asymmetry when fewer studies are included.

#### Quality Assessment

The quality of included clinical trials was assessed using either Cochrane risk-of-bias tool for randomized trials (RoB2) [27] or Risk Of Bias In Non-randomized Studies—of Interventions (ROBINS-I) tool [28] based on the design of each trial. The domains of the suitable tool were assessed for each included study by two authors independently, and the lead author resolved conflicts. The five domains of RoB2 domains were assessed for each randomized clinical trial, including (1) randomization process, (2) deviations from intended interventions, (3) missing outcome data, (4) measurement of the outcome and (5) selection of the reported result. The signaling questions were answered as yes (Y), probably yes (PY), no (N), probably no (PN) or no information (NI). Bias in each domain was judged accordingly as high risk, low risk or some concerns. The seven domains of ROBINS-I were also assessed for each non-randomized clinical trial by two independent authors as well, including (1) bias due to confounding, (2) bias in the selection of participants, (3) bias in classification of the intervention, (4) bias due to deviations from intended interventions, (5) bias due to missing outcome data, (6) bias in measurement of the outcome, and (7) bias in selection of the reported results.

## 3. Results

### 3.1. Data Collection and Study Selection

**Figure 1:**
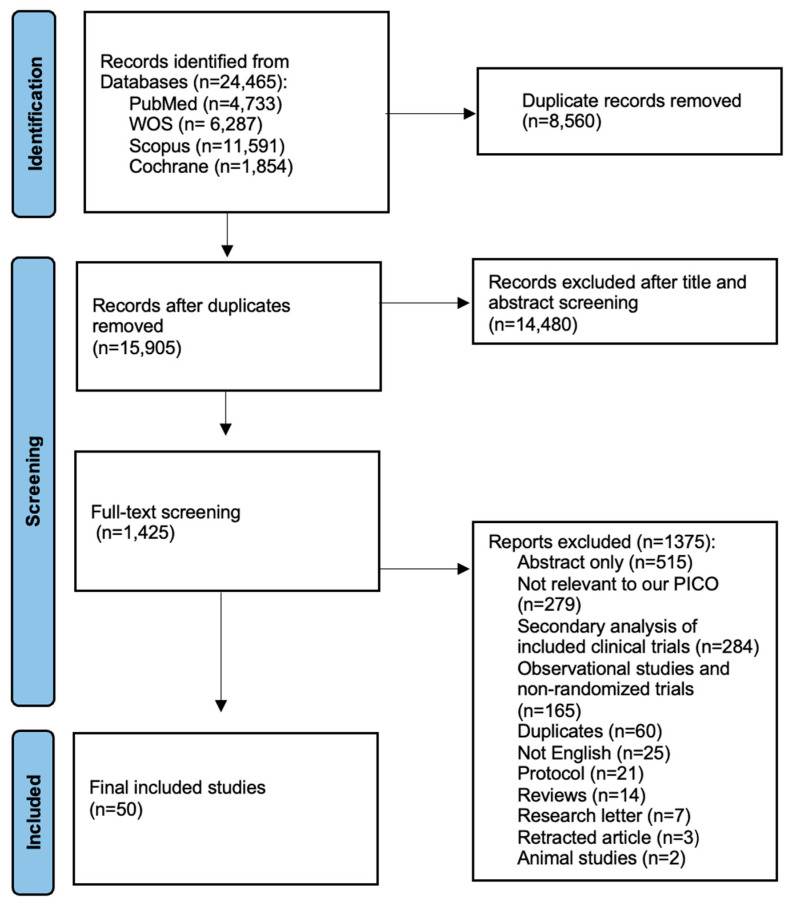
PRISMA flow diagram.

Our systematic search retrieved 24,465 results from different databases, which was reduced to 15,905 records after duplicate records were removed. After screening of the titles and abstracts of the 15,905 records, only 1425 were eligible for full-text screening. Finally, 50 reports were included after data synthesis [15,16,17,29,30,31,32,33,34,35,36,37,38,39,40,41,42,43,44,45,46,47,48,49,50,51,52,53,54,55,56,57,58,59,60,61,62,63,64,65,66,67,68,69,70,71,72,73,74,75]. The detailed selection process is illustrated in the PRISMA flow diagram (Figure 1).

### 3.2. Characteristics of the Included Studies

All of the included studies were clinical trials with 50 randomized controlled trials [15,16,17,29,30,31,32,33,34,35,36,37,38,39,40,41,42,43,44,45,46,47,48,49,50,51,52,53,54,55,56,57,58,59,60,61,62,63,64,65,66,67,68,69,70,71,72,73,74]. The included studies adopted various SGLT2 inhibitors including dapagliflozin (n = 24) [17,36,38,39,40,41,44,46,52,53,54,56,57,58,59,61,63,64,67,71,72,75], empagliflozin (n = 17) [16,29,31,34,37,45,49,50,51,55,60,65,66,68,69,70,73], canagliflozin (n = 2) [43,62], sotagliflozin (n = 2) [32,33], tofogliflozin (n = 2) [42,47], ertugliflozin (n = 1) [35], ipragliflozin (n = 1) [74] and janagliflozin (n = 1) [52]. Patients in one study adopted various SGLT2 inhibitors instead of a single drug, with no outcome data regarding each drug separately [48].

The included studies included diverse populations including acute cardiac decompensation (n = 17) [29,33,36,37,38,40,44,53,55,58,65,68,69,70,72,75], chronic heart failure (n = 11) [31,34,45,51,54,56,57,59,60,61,64,67], DM or high-risk DM (n = 8) [42,43,46,47,49,52,71,74], DM with established CVD (n = 4) [35,50,73], CKD (n = 6) [30,32,39,62,68], COVID-19 (n = 3) [15,16,48], cardiac procedures (n = 2) [63,66], critically ill patients (n = 1) [17] and functional mitral regurgitation (MR) (n = 1) [41]. Summaries of the included studies and baseline characteristics are provided in Table 1 and Table 2, respectively. The reported comorbidities of the population of the included studies are presented in Supplementary Table S2.

### 3.3. Quality Assessment

**Figure 2:**
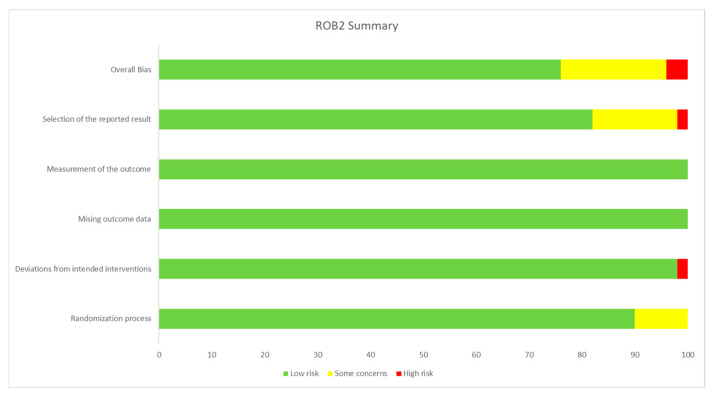
Risk of Bias 2 (RoB2) summary.

The quality of the included RCTs was assessed using the RoB2 tool. Most of the included RCTs showed a low risk of bias; however, ten studies raised some concerns [29,36,37,43,50,53,55,58,64,72], and two studies showed a high risk of bias [40,74]. Of the ten trials that raised some concerns, four trials raised concerns regarding the randomization process [36,37,43,58], and eight trials raised concerns regarding the selection of the reported results [29,36,50,53,55,58,64,72]. Of the two trials that showed a high risk of bias, one raised some concerns regarding the randomization process and showed a high risk of bias regarding the domain of deviations from the intended outcome [40]. The other one showed a high risk of bias regarding the selection of the reported results [74]. The RoB2 summary is shown in Figure 2, while the detailed RoB2 figure for the 50 trials is provided in Supplementary Figure S1.

### 3.4. Mortality Outcomes

#### 3.4.1. Mortality (Up to One Year)

Of the included studies, 34 studies [15,16,30,33,36,37,38,40,41,42,43,44,45,46,48,49,50,51,52,53,55,56,58,59,63,64,65,66,69,70,72,74,75] reported the outcome of mortality up to one year. The SGLT2 inhibitors group included 10,981 patients, while the control group included 9979 patients. The pooled risk ratio showed that SGLT2 inhibitors significantly reduced mortality as compared to the control group (RR = 0.89, 95% CI [0.80, 0.99], *p* = 0.03). The included studies showed no heterogeneity (I^2^ = 0%, *p* = 0.57) (Supplementary Figure S2). This significant short-term mortality benefit was maintained in a sensitivity analysis excluding the two trials with a high risk of bias (Heshmat et al. 2025 and Kaku et al. 2019) [40,74]. Kaku et al. 2019 [74] reported zero events in both arms and did not contribute statistical weight, while the exclusion of Heshmat et al. 2025 [40] did not alter the pooled estimates, which confirms that our findings are driven by high-quality RCTs (Supplementary Figure S3). In a meta-regression model, mortality up to one year showed no significant correlation with baseline weight or BMI mean difference (*p* = 0.630 and 0.603, respectively).

Visual inspection of the funnel plot for mortality up to one year revealed a symmetrical distribution of the included studies. Furthermore, Egger’s regression test indicated no significant small-study effects or publication bias (*p* = 0.734) (Supplementary Figure S4).

In a subgroup analysis based on study population, 14 studies [33,36,37,38,40,44,53,55,58,65,69,70,72,75] were included in the subgroup of acute cardiac decompensation, while the subgroup of chronic heart failure included five studies [45,51,56,59,64]. SGLT2 inhibitors significantly reduced mortality in the subgroup of acute cardiac decompensation (RR = 0.76, 95% CI [0.60, 0.97], *p* = 0.03). However, no significant difference was observed among the groups in the chronic heart failure group (RR = 1.65, 95% CI [0.57, 4.80], *p* = 0.36). No heterogeneity was observed in either subgroup (I^2^ = 14%, *p* = 0.3; I^2^ = 0%, *p* = 0.88, respectively). Two studies [45,64] of the five studies included in the subgroup of chronic heart failure showed no events in either the SGLT2 inhibitor or control groups, with no estimable RRs for them. The subgroup of DM/high-risk DM included six studies [42,43,46,49,52,74], while the subgroup of DM with established CVD included only one study [50]. Both subgroups showed no significant difference between the two groups (RR = 1.98, 95% CI [0.22, 17.59], *p* = 0.54; RR = 0.63, 95% CI [0.20, 1.19], *p* = 0.41, respectively). Five studies [42,43,46,52,74] of the six studies included in the subgroup of DM/high-risk DM showed no events in both study groups; therefore, the RRs of these studies were not estimable.

The subgroup of COVID-19/critically ill patients included three studies [15,16,48] and showed no significant difference between the two groups as well (RR = 0.93, 95% CI [0.81, 1.06], *p* = 0.27). Two studies [63,66] were included in the subgroup of cardiac procedures, with no significant difference between the two groups (RR = 0.89, 95% CI [0.61, 1.29], *p* = 0.53). Only one study [41] was included in the subgroup of functional MR, and another one [30] was included in subgroup of CKD. The RR of each one showed no significant difference between the two groups (RR = 2.00, 95% CI [0.19, 21.38], *p* = 0.57; RR = 6.90, 95% CI [0.36, 132.85], *p* = 0.20, respectively) (Supplementary Figure S5).

Another subgroup analysis was performed based on the SGLT2 inhibitor adopted in each study. The subgroups of dapagliflozin and empagliflozin included 16 [36,38,40,41,44,46,48,52,53,56,58,59,63,64,72,75] and 12 studies [16,30,37,45,49,50,51,55,65,66,69,70], respectively. The pooled RR of the dapagliflozin subgroup significantly favored it over the control (RR = 0.83, 95% CI [0.69, 1.00], *p* = 0.05). However, analysis of the empagliflozin subgroup did not favor either the SGLT2 inhibitor group or the control group (RR = 0.84, 95% CI [0.51, 1.38], *p* = 0.14). Three [46,52,64] and two [45,65] studies in the dapagliflozin and empagliflozin subgroups, respectively, showed no events in either study group; therefore, no estimable RRs were available for them. Both subgroups showed no evident heterogeneity (I^2^ = 0%, *p* = 0.81; I^2^ = 34%, *p* = 0.14, respectively).

Sotagliflozin subgroup included only one study [33], and its RR did not favor either of the two groups (RR = 0.86, 95% CI [0.63, 1.18], *p* = 0.36). The subgroup of multiple SGLT2 inhibitors also included one study [48], and its RR showed no significant difference between the two groups (RR = 0.89, 95% CI [0.59, 1.34], *p* = 0.56). Canagliflozin [43], ipragliflozin [74], janagliflozin [52] and tofogliflozin [42] included only one study each with zero events in either the intervention or the control; therefore, the risk ratio was not estimable (Supplementary Figure S6).

#### 3.4.2. Mortality (More than One Year)

**Figure 3:**
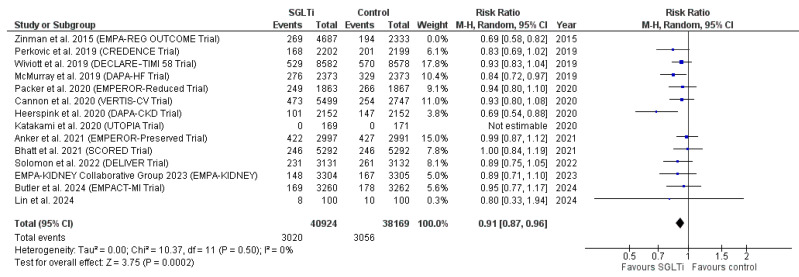
Sensitivity analysis of all-cause mortality beyond one year [31,32,34,35,39,47,54,57,60,62,67,68,71,73].

Of the included studies, 14 studies [31,32,34,35,39,47,54,57,60,62,67,68,71,73] reported the outcome of mortality more than one year. The SGLT2 inhibitors group included 45,611 patients, while the control group included 40,502 patients. The pooled risk ratio favored the SGLT2 inhibitors group over the control group (RR = 0.89, 95% CI [0.85, 0.94], *p* < 0.0001). The included studies were heterogeneous (I^2^ = 37%, *p* = 0.08); however, heterogeneity was resolved after removing the results of Zinman et al. 2015 (EMPA-REG OUTCOME Trial) [73] (I^2^ = 29%, *p* = 0.14). The pooled RR remained in favor of SGLT2 inhibitors after the leave-one-out test (RR = 0.91, 95% CI [0.87, 0.96], *p* = 0.0002) (Figure 3).

Visual inspection of the funnel plot for mortality more than one year revealed a symmetrical distribution of the included studies. Furthermore, Egger’s regression test indicated no significant small-study effects or publication bias (*p* = 0.1994) (Supplementary Figure S7).

In a subgroup analysis based on population, four studies [32,39,62,68] were included in the subgroup of CKD, and the pooled RR of the subgroup favored the SGLT2 inhibitors subgroup over the control group (RR = 0.86, 95% CI [0.74, 0.99], *p* = 0.004). The pooled studies within the subgroup were homogeneous (I^2^ = 52%, *p* = 0.1). In a meta-regression model, no significant correlation was observed between mortality more than one year and baseline BMI mean difference among patients with CKD.

Five studies [31,54,57,60,67] were included in the subgroup of chronic heart failure, while only one study [34] was included in the subgroup of acute cardiac decompensation. The pooled RR of the subgroup of chronic heart failure favored the SGLT2 inhibitors group over the control group (RR = 0.92, 95% CI [0.85, 0.99], *p* = 0.02), with no heterogeneity (I^2^ = 0%, *p* = 0.56), while the RR of the acute cardiac compensation study did not favor either of the two groups (RR = 0.95, 95% CI [0.77, 1.17], *p* = 0.62).

**Figure 4:**
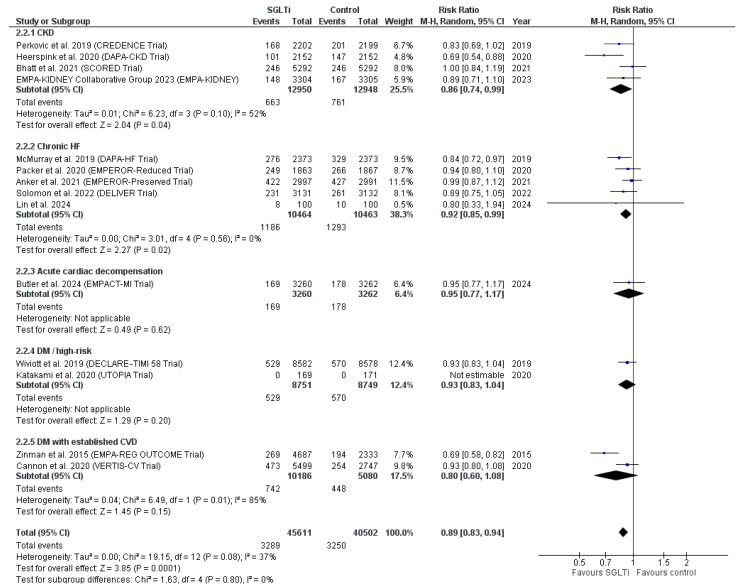
Subgroup analysis based on population with sensitivity analysis of the subgroup of DM with established CVD [31,32,34,35,39,47,54,57,60,62,67,68,71,73].

Each of the subgroups of DM/high-risk DM [47,71] and DM with established CVD included two studies [35,73]. One of the studies in the DM/high-risk DM subgroup showed no events in either study group; therefore, it showed no estimable RR [47]. The RR of the other study showed no significant difference between the two groups (RR = 0.93, 95% CI [0.83, 1.04], *p* = 0.2). The pooled RR of the subgroup of DM with established CVD showed no significant difference between the two groups (RR = 0.80, 95% CI [0.60, 1.08], *p* = 0.15), with high heterogeneity (I^2^ = 85%, *p* = 0.01) (Figure 4).

Another subgroup analysis was performed based on the adopted SGLT2 inhibitor in each study. Five studies [39,54,57,67,71] were included in the dapagliflozin subgroup, and another five studies [31,34,60,68,73] were included in the empagliflozin subgroup. The pooled RR of the dapagliflozin subgroup favored the SGLT2 inhibitors group over the control group, while the pooled RR of the empagliflozin subgroup did not favor either of the two groups (RR = 0.86, 95% CI [0.79, 0.94], *p* = 0.001; RR = 0.89, 95% CI [0.78, 1.01], *p* = 0.07, respectively). The dapagliflozin subgroup showed no heterogeneity, while heterogeneity was evident in the empagliflozin subgroup (I^2^ = 21%, *p* = 0.28; I^2^ = 64%, *p* = 0.02, respectively). Heterogeneity was best resolved in the empagliflozin subgroup after removing Zinman et al. 2015—EMPA-REG OUTCOME trial [73] (I^2^ = 0%, *p* = 0.86). The pooled RR did not favor either of the two groups after the leave-one-out test (RR = 0.95, 95% CI [0.88, 1.03], *p* = 0.25).

**Figure 5:**
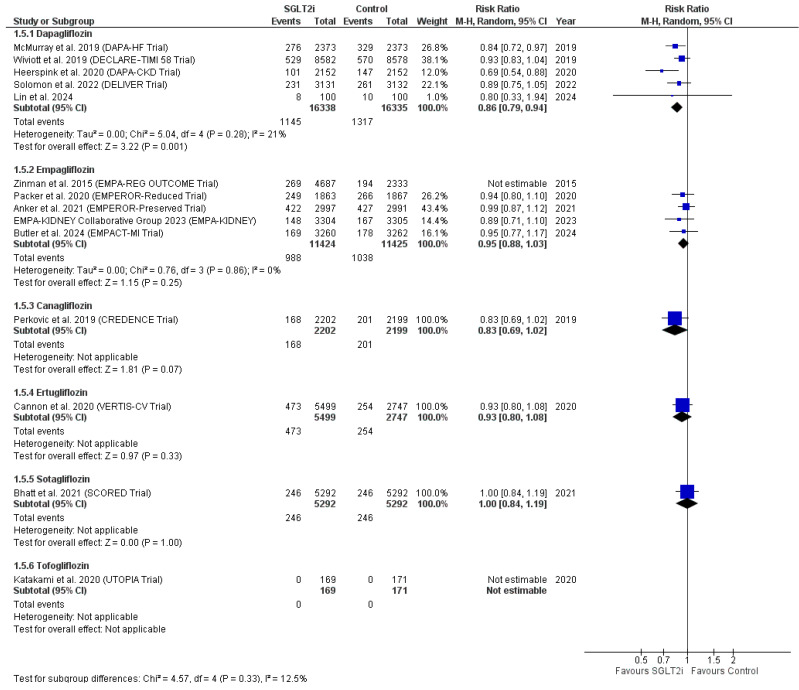
Subgroup analysis based on the adopted SGLT2 inhibitors with sensitivity analysis of the subgroup of empagliflozin [31,32,34,35,39,47,54,57,60,62,67,68,71,73].

The subgroups of canagliflozin [62], ertugliflozin [35] and sotagliflozin [32] included only one study each, with a risk ratio that did not favor either of the two groups in each subgroup (RR = 0.83, 95% CI [0.69, 1.02], *p* = 0.07; RR = 0.93, 95% CI [0.80, 1.08], *p* = 0.33; RR = 1.00, 95% CI [0.84, 1.19], *p* = 1.00, respectively). The subgroup of tofogliflozin also included one study only [47], but it showed no events in either of the study groups, with no estimable RR (Figure 5).

### 3.5. Cardiovascular Mortality

The outcome of cardiovascular mortality was reported by 25 studies [29,31,32,33,34,35,37,39,41,44,47,50,51,57,59,60,61,62,63,67,68,70,71,73,75], with a pooled RR that favored the SGLT2 inhibitor group over the control group (RR = 0.88, 95% CI [0.84, 0.94], *p* < 0.0001). No heterogeneity was observed among the included studies (I^2^ = 0%, *p* = 0.51) (Supplementary Figure S8). In a meta-regression model, cardiovascular mortality showed no significant correlation with baseline BMI mean difference (*p* = 0.536).

Visual inspection of the funnel plot for cardiovascular mortality revealed a symmetrical distribution of the included studies. Furthermore, Egger’s regression test indicated no significant small-study effects or publication bias (*p* = 0.778) (Supplementary Figure S9).

In a subgroup analysis based on population, seven studies [29,33,34,37,44,70,75] were included in the subgroup of acute cardiac compensation, while the subgroup of chronic heart failure included seven studies [31,51,57,59,60,61,67]. The pooled RR of the subgroup of acute cardiac decompensation did not favor either of the two groups, while the pooled RR of the subgroup of chronic heart failure favored the SGLT2 inhibitors group over the control group (RR = 0.96, 95% CI [0.81, 1.14], *p* = 0.61; RR = 0.91, 95% CI [0.84, 0.98], *p* = 0.01, respectively). Both subgroups showed no heterogeneity between studies (I^2^ = 0%, *p* = 0.71; I^2^ = 0%, *p* = 0.82, respectively).

The subgroup of DM/high-risk DM included two studies [47,71], while the subgroup of DM with established CVD included three studies [35,50,73]. The pooled RR of both subgroups did not favor either of the two groups (RR = 0.98, 95% CI [0.83, 1.17], *p* = 0.85; RR = 0.75, 95% CI [0.52, 1.06], *p* = 0.11, respectively). One of the two studies in the subgroups of DM/high-risk DM reported no events in either study group, with no estimable RR [47]. The subgroup of DM with established CVD showed high heterogeneity (I^2^ = 75%, *p* = 0.02), which was best resolved after removing Cannon et al. 2020 (VERTIS-CV Trial) (I^2^ = 0%, *p* = 0.75) [35]. The pooled RR after the leave-one-out test favored the SGLT2 inhibitors group over the control group (RR = 0.62, 95% CI [0.50, 0.77], *p* < 0.0001).

The subgroup of CKD included four studies [32,39,62,68] with a pooled RR that favored the SGLT2 inhibitors group over the control group (RR = 0.85, 95% CI [0.74, 0.97], *p* = 0.01). No heterogeneity was observed within the subgroup (I^2^ = 0%, *p* = 0.82). In a meta-regression model, no significant correlation was observed between cardiovascular mortality and baseline BMI mean difference among patients with CKD (*p* = 0.787). The subgroups of cardiac procedures [63] and functional MR [41] included only one study each, with pooled RRs that did not favor either SGLT2 inhibitors or control (RR = 0.83, 95% CI [0.51, 1.37], *p* = 0.47; RR = 2.00, 95% CI [0.19, 21.38], *p* = 0.57, respectively) (Supplementary Figure S10).

Another subgroup analysis was performed based on the SGLT2 inhibitor adopted by each study. The dapagliflozin subgroup included ten studies [39,41,44,57,59,61,63,67,71,75], while the empagliflozin subgroup included ten studies [29,31,34,37,50,51,60,68,70,73]. The pooled RR of both subgroups favored the SGLT2 inhibitors group over the control group (RR = 0.92, 95% CI [0.85, 0.99], *p* = 0.03; RR = 0.85, 95% CI [0.73, 0.98], *p* = 0.03, respectively). Both subgroups showed no heterogeneity between studies (I^2^ = 0%, *p* = 0.85; I^2^ = 36%, *p* = 0.12, respectively).

The subgroups of canagliflozin [62] and ertugliflozin [35] included only one study each, while the sotagliflozin subgroup included two studies [32,33], with a risk ratio that did not favor either of the two groups in each subgroup (RR = 0.78, 95% CI [0.62, 1.00], *p* = 0.05; RR = 0.93, 95% CI [0.78, 1.10], *p* = 0.38; RR = 0.91, 95% CI [0.75, 1.09], *p* = 0.29, respectively). The subgroup of tofogliflozin also included one study only, but it showed no events in either of the study groups, with no estimable RR [47] (Supplementary Figure S11).

### 3.6. In-Hospital Mortality

Five included studies [17,36,38,48,58] reported the outcome of in-hospital mortality. The pooled RR did not favor either of the two groups (RR = 1.03, 95% CI [0.84, 1.28], *p* = 0.76), with no heterogeneity between studies (I^2^ = 0%, *p* = 0.58) (Supplementary Figure S12).

In a subgroup analysis based on the population who received the treatment, three studies were included in the subgroup of acute cardiac decompensation [36,38,58], while two studies were included in the subgroup of COVID-19/critical illness [17,48]. The pooled RR of both subgroups did not favor either SGLT2 inhibitors or the control (RR = 0.63, 95% CI [0.16, 2.54], *p* = 0.52; RR = 1.05, 95% CI [0.84, 1.30], *p* = 0.66, respectively), with no heterogeneity between studies within each subgroup (I^2^ = 27%, *p* = 0.24; I^2^ = 0%, *p* = 0.73, respectively) (Supplementary Figure S13).

Another subgroup analysis was performed based on the adopted SGLT2 inhibitor in each study. Four studies adopted dapagliflozin [17,36,38,58], while one study adopted multiple SGLT2 inhibitors [48]. The pooled RR did not favor the SGLT2 inhibitor group or the control group in either subgroup (RR = 1.02, 95% CI [0.80, 1.28], *p* = 0.90; RR = 1.14, 95% CI [0.67, 1.95], *p* = 0.63, respectively). The dapagliflozin subgroup showed no heterogeneity (I^2^ = 0%, *p* = 0.40) (Supplementary Figure S14).

#### Renal Mortality

Three studies reported the outcome of renal mortality [35,39,62]. The pooled RR did not favor either of the study groups (RR = 0.37, 95% CI [0.12, 1.14], *p* = 0.08). The outcome showed no heterogeneity between the included studies (I^2^ = 0%, *p* = 0.88) (Supplementary Figure S15). One of the included studies showed no events in either of the two groups, with no estimable RR [35]. The other two studies included patients with CKD: one of them adopted dapagliflozin [39], and the other adopted canagliflozin [62].

## 4. Discussion

The current systematic review and meta-analysis aims to provide a comprehensive review, analyzing the effect of SGLT2 inhibitors on mortality outcomes across different clinical populations. In addition to the overall pooled results of the included studies, the adoption of subgroup analyses allowed us to provide separate pooled results for each population including acute cardiac compensation, chronic heart failure, CKD, DM/high-risk DM, DM with established CVD, COVID-19/critically ill patients, cardiac procedures and functional MR. The subgroup analyses also allowed for synthesizing the pooled results for each SGLT2 inhibitor medication separately including dapagliflozin, empagliflozin, canagliflozin, ertugliflozin, janagliflozin, sotagliflozin, and tofogliflozin.

A major finding of the current updated meta-analysis is that SGLT2 inhibitors significantly reduce all-cause mortality within the first year of treatment (RR = 0.89, 95% CI [0.80, 0.99], *p* = 0.03). Subgroup analysis revealed that this early survival benefit is predominantly driven by patients receiving SGLT2 inhibitors in the setting of acute cardiac decompensation (RR = 0.76, 95% CI [0.60, 0.97], *p* = 0.03).

Similarly, the outcome of all-cause mortality more than one year showed consistent reduced mortality rates among patients who received SGLT2 inhibitors compared to control patients (RR = 0.89, 95% CI [0.85, 0.94], *p* < 0.0001). The consistency between the short-term and long-term outcomes could be explained by the immediate hemodynamic, diuretic, and natriuretic relief contributing to early survival in acute settings, followed by cumulative cardio-protective and reno-protective effects that sustain survival over the years [76].

A previously published meta-analysis reported that SGLT2 inhibitors significantly reduced all-cause mortality (hazard ratio (HR) = 0.86, 95% CI [0.80, 0.93]) [77]. Also, another systematic review and meta-analysis reported that SGLT2 inhibitors significantly reduced all-cause mortality among various populations (RR = 0.89, 95% CI [0.84, 0.95], *p* = 0.0006) [18].

While large cardiovascular outcome trials have established SGLT2 inhibitors as foundational therapies for heart failure and chronic kidney disease, individual trials are typically powered for composite endpoints rather than isolated mortality. The current meta-analysis provides incremental knowledge beyond these existing trials in several ways. First, by synthesizing data from 50 trials involving over 85,000 patients in recent years, we provide a highly powered and definitive quantification of mortality reduction as a standalone clinical endpoint. Second, our temporal stratification of up to one year versus beyond one year introduces a crucial clinical insight. It demonstrates that the survival benefits of SGLT2 inhibitors are not strictly dependent on long-term structural disease modification or cumulative renoprotection. Instead, our findings highlight a distinct early survival benefit that is particularly driven in patients with acute cardiac decompensation. This early divergence in mortality curves strongly reinforces recent shifts in clinical practice advocating the prompt in-hospital initiation of SGLT2 inhibitors during acute vulnerability rather than deferring treatment to the stable outpatient setting.

Synthesizing cardiovascular mortality outcomes in the current study revealed a significantly lower cardiovascular mortality among patients who received SGLT2 inhibitors compared to control patients (RR = 0.88, 95% CI [0.84, 0.94], *p* < 0.0001). A previously published meta-analysis reported a significantly lower cardiovascular mortality among diverse populations (HR = 0.8, 95% CI [0.78, 0.92]) [77]. Another more recent meta-analysis confirmed the same results, which aligned with our findings [18]. The lower cardiovascular mortality rates are attributed to the cardio-protective impact of SGLT2 inhibitors, such as decreasing preload and afterload and improving endothelial function and left ventricular function [78].

Our findings also revealed that patients who suffered from DM with established CVD showed significantly lower cardiovascular mortality (RR = 0.62, 95% CI [0.50, 0.77], *p* < 0.0001) following sensitivity analysis to resolve heterogeneity. These findings support the current guideline recommendations to adopt SGLT2 inhibitors for patients with atherosclerotic cardiovascular disease and those at high risk of cardiovascular diseases [6]. Conversely, patients who suffered from DM/high-risk DM showed no significant difference regarding cardiovascular mortality (RR = 0.98, 95% CI [0.83, 1.17], *p* = 0.85). The lack of significant difference regarding cardiovascular mortality can be attributed to the lack of statistical power or the competing risks of non-cardiovascular deaths. However, this insignificant difference warrants cautious interpretations and highlights the need for RCTs that study the effect of SGLT2 inhibitors on mortality outcomes among diabetic patients with different background illnesses.

Among patients with chronic heart failure, those who received SGLT2 inhibitors showed significantly lower all-cause mortality beyond one-year follow-up and significantly lower cardiovascular mortality (RR = 0.92, 95% CI [0.85, 0.99], *p* = 0.02 and RR = 0.91, 95% CI [0.84, 0.98], *p* = 0.01, respectively). These findings go in alignment with the current recommendations of ESC 2021, the ESC 2023 update and the ACC/AHA/HFSA 2022 guidelines to consider SGLT2 inhibitors a class I therapy, especially for HFrEF [7,8,9].

While SGLT2 inhibitors demonstrate robust mortality benefits across the general heart failure spectrum, their disease-modifying efficacy appears to be attenuated in patients with advanced heart failure. A recent study by Nuzzi et al. compared the initiation of SGLT2 inhibitors in advanced versus non-advanced HFrEF outpatients [79]. The authors found that while the drugs were highly tolerated and safe regarding kidney function, patients with advanced heart failure did not experience the significant improvements in NT-proBNP levels, left ventricular ejection fraction (LVEF), or NYHA functional class that were observed in the non-advanced cohort. This blunted response in the advanced stages is likely due to diminished biological reserves, extensive myocardial fibrosis, and extreme baseline neurohormonal activation that resists further pharmacological modulation [79]. These observational findings underscore the critical importance of early therapy initiation, as highlighted by our short-term mortality findings, to maximize survival benefits before irreversible cardiac structural alterations and end-stage disease progression occur.

Among patients with CKD, those who received SGLT2 inhibitors showed significantly lower all-cause mortality beyond one-year follow-up and significantly lower cardiovascular mortality as well (RR = 0.86, 95% CI [0.74, 0.99], *p* = 0.004 and RR = 0.85, 95% CI [0.74, 0.97], *p* = 0.01, respectively). These findings support the recent recommendation by the KDIGO 2024 guidelines to consider SGLT2 inhibitors for any adult patient with CKD, irrespective of glycemic status [13]. Meta-regression models have been conducted in the current study to explore the potential correlation between baseline weight and BMI mean difference and mortality outcomes. All of the conducted meta-regression models showed insignificant correlations including the meta-regression model that was specified for patients with CKD in correlation with baseline BMI mean difference. Future studies should consider reporting the baseline characteristics of patients with CKD, including weight, BMI and sarcopenic obesity, to allow for a comprehensive meta-regression that explores how these baseline variables can influence the impact of SGLT2 inhibitors on mortality outcomes.

The pooled results of dapagliflozin in all-cause mortality beyond one year and cardiovascular mortality were significantly in favor of dapagliflozin (RR = 0.86, 95% CI [0.79, 0.94], *p* = 0.001; RR = 0.92, 95% CI [0.85, 0.99], *p* = 0.03, respectively). Furthermore, dapagliflozin also demonstrated a significant reduction in short-term mortality up to one year (RR = 0.83, 95% CI [0.69, 1.00], *p* = 0.05). The findings of all-cause mortality align with the meta-analysis of Mukhopadhyay et al. 2022 [77], where dapagliflozin significantly differed from the control group (HR = 0.83, 95% CI [0.72, 0.97]. On the other hand, our findings regarding dapagliflozin in cardiovascular mortality contradict the findings of Mukhopadhyay et al. 2022, where dapagliflozin did not offer better outcomes compared to the control (HR = 0.88, 95% CI [0.78, 1.00]) [77].

In the current study, empagliflozin offered no significant difference compared to the control regarding all-cause mortality (RR = 0.89, 95% CI [0.78, 1.01], *p* = 0.07); however, it showed significantly lower cardiovascular mortality rates (RR = 0.85, 95% CI [0.73, 0.98], *p* = 0.03). The insignificant results regarding empagliflozin in all-cause mortality align with the findings of Mukhopadhyay et al. 2022 (HR = 0.86, 95% CI [0.69, 1.08]; however, the significantly lower cardiovascular mortality rates in our meta-analysis contradict those of Mukhopadhyay et al. 2022 (HR = 0.81, 95% CI [0.63, 1.03]) [77]. The rest of the included SGLT2 inhibitors showed a very limited number of studies, with minimal statistical power, which may be responsible for the insignificant findings.

### Strengths and Limitations

The current systematic review offers a comprehensive, up-to-date evaluation of the literature related to the impact of SGLT2 inhibitors on mortality outcomes, incorporating meta-analysis and subgroup analyses. A major strength of this updated study is the strict exclusion of observational and non-randomized post hoc data, which ensured the strict inclusion of RCTs. Most of the included RCTs were judged as having a low risk of bias using RoB2, provided by Cochrane, which constitutes a major strength of the current work. In addition, the inclusion of diverse populations and different SGLT2 inhibitors allowed for comparison of the benefits of these interventions across various populations. In most cases, heterogeneity, when evident, was successfully resolved after leave-one-out analysis. Meta-regression was also performed as a statistical tool to explore the effect of baseline weight and BMI mean differences on mortality outcomes.

Despite these strengths, several limitations were apparent. The limited number of studies examining canagliflozin, ertugliflozin, janagliflozin, sotagliflozin, and tofogliflozin hindered the ability to draw specific conclusions and provide tailored recommendations for these interventions. Furthermore, certain populations, such as patients with structural cardiac abnormalities and those with critical illness, were rarely examined in the available clinical trials. Additionally, despite subgrouping by underlying baseline pathology, pooling diverse clinical scenarios inherently introduces clinical heterogeneity, and overall pooled estimates must be interpreted alongside disease-specific subgroup findings. Therefore, well-designed randomized controlled trials are recommended in the future to better evaluate less-studied SGLT2 inhibitors and under-represented populations.

## 5. Conclusions

The present strictly RCT-based systematic review and meta-analysis demonstrates that SGLT2 inhibitors, as a class, provide a consistent and significant reduction in both all-cause and cardiovascular mortality. Crucially, this survival benefit is evident not only during long-term follow-up for patients with chronic heart failure, chronic kidney disease (CKD), and high-risk diabetes mellitus but also in the short term (up to one year). The early mortality reduction is particularly pronounced when SGLT2 inhibitors are promptly initiated during acute cardiac decompensation. Furthermore, cardiovascular mortality benefits are evident across multiple clinical profiles, though the magnitude of the effect varies across subgroups and specific pharmacological agents. Future well-designed RCTs are needed to address the impact of less-explored SGLT2 inhibitors and understudied populations.

## Figures and Tables

**Table 1:** Summary of the included studies.

Study ID (Trial Name)	Study Design	Country/Region	Population	Intervention	Control	Total Sample Size	Follow-Up Duration
Berg et al. 2025 (DAPA ACT HF-TIMI 68 Trial) [75]	RCT	Multi-national (North America, Europe)	Acute heart failure	Dapagliflozin: 10 mg/d	Placebo	2401	2 months
Agarwal et al. 2025 [30](CONFIDENCE Trial)	RCT	Multi-national (Europe, Asia, North America)	Chronic kidney disease with type 2 diabetes	Empagliflozin: 10 mg/d Finerenone: 10 or 20 mg/d	Finerenone 10 or 20 mg/d	533	7.0 months
Heshmat et al. 2025 [40]	RCT	Egypt	Acute myocardial infarction (STEMI) with diabetes	Dapagliflozin: 10 mg/d	Standard therapy	54	1 month
Huang et al. 2025 [41] (DEFORM Trial)	RCT	China	Functional mitral regurgitation	Dapagliflozin: 10 mg/d	Guideline-directed medical therapy	104	3 months
Mocan et al. 2025 [58]	RCT	Romania	Acute heart failure	Dapagliflozin: 10 mg/d + structured intravenous furosemide	Structured intravenous furosemide	98	1 month
Raposeiras-Roubin et al. 2025 (DapaTAVI Trial) [63]	RCT	Spain	Transcatheter Aortic Valve Implantation with a prior episode of aortic stenosis-related heart failure	Dapagliflozin: 10 mg/d	Standard therapy	1222	12 months
Snel et al. 2025 [66]	RCT	The Netherlands	Elective cardiopulmonary bypass (CPB) assisted cardiac surgery	Empagliflozin: 10 mg/d	Standard perioperative care	55	1 week
Zhou et al. 2025 [72]	RCT	China	Acute myocardial infarction with early heart failure	Dapagliflozin: 10 mg/d	Standard therapy	98	12 months
Butler et al. 2024 [34](EMPACT-MI Trial)	RCT	Multi-national (South America, Europe, Asia, North America, Australia)	High-risk patients with acute myocardial infarction	Empagliflozin: 10 mg/d	Placebo	6522	17.9 months
James et al. 2024 (DAPA-MI Trial) [44]	RCT	Sweden, United Kingdom	Acute myocardial infarction	Dapagliflozin: 10 mg/d	Placebo	4017	11.6 months (IQR 6.8–16.9; max 29)
Kosiborod et al. 2024 [48] (ACTIV-4a Trial)	RCT	Multi-national (South America, Europe, North America)	COVID-19 hospitalized population	Dapagliflozin: 10 mg/d, Empagliflozin: 10 mg/d, Canagliflozin: 100 mg/d or Ertugliflozin: 5 mg/d	Standard therapy	573	3.0 months
Kumar et al. 2024 [50]	RCT	India	Type 2 diabetes with established cardiovascular disease	Empagliflozin: 10 or 25 mg/d	Placebo	250	1.0 month
Li et al. 2020 [52]	RCT	China	Type 2 diabetes patients following healthcare provider guidance around diet and physical exercise or using metformin therapy	Dapagliflozin: 10 mg/d Janagliflozin: 25 or 50 mg/d	Placebo	36	2.9 weeks
Liang et al. 2024 [53]	RCT	China	Acute myocardial infarction with new-onset HF	Dapagliflozin (an initial dosage of 5 mg/d, which was then increased to 10 mg/d) + sacubitril/valsartan	Standard therapy + sacubitril/valsartan	60	6 months
Lin et al. 2024 [54]	RCT	China	Chronic heart failure (HFrEF with hyperuricemia)	Dapagliflozin: 10 mg/d	Placebo	200	24 months
McMurray et al. 2024 [56] (DETERMINE-preserved Trial)	RCT	Multi-national (South America, Europe, Asia, Africa, North America)	Chronic heart failure (HFpEF)	Dapagliflozin: 10 mg/d	Placebo	504	3.7 months
McMurray et al. 2024 [56] (DETERMINE-reduced Trial)	RCT	Multi-national (South America, Europe, North America)	Chronic heart failure (HFrEF)	Dapagliflozin: 10 mg/d	Placebo	313	3.7 months
Pastore et al. 2024 [61] (DAPA-ECHO Trial)	RCT	Italy	Chronic heart failure (non-diabetic HFrEF/HFmrEF)	Dapagliflozin: 10 mg/d	Optimal Medical Therapy	88	6 months
Tavares et al. 2024 [17] (DEFENDER Trial)	RCT	Brazil	Critical illness/ICU population	Dapagliflozin: 10 mg/d	Standard therapy	507	4 weeks
Emara et al. 2023 [38] (DAPA-RESPONSE-AHF Trial)	RCT	Egypt	Acute heart failure	Dapagliflozin: 10 mg/d	Placebo	87	2.0 months
Liu et al. 2023 [55]	RCT	China	Acute heart failure	Empagliflozin: 10 mg/d	Standard therapy	105	2.0 months
RECOVERY Collaborative Group 2023 (RECOVERY Trial) [16]	RCT	Multi-national (Asia, Africa)	COVID-19 hospitalized population	Empagliflozin: 10 mg/d	Usual therapy	4271	4 weeks
EMPA-KIDNEY Collaborative Group 2023 (EMPA-KIDNEY) [68]	RCT	Multi-national (Asia, Europe, North America)	Chronic kidney disease (with or without diabetes)	Empagliflozin: 10 mg/d	Placebo	6609	24.0 months (IQR 18.0–28.8)
Adel et al. 2022 [29]	RCT	Iran	Acute coronary syndrome with diabetes	Empagliflozin: low dose	Placebo	93	6 months
Charaya et al. 2022 [36]	RCT	Russia	Acute heart failure	Dapagliflozin: 10 mg/d	Standard therapy	102	1 month
Reis et al. 2022 [64]	RCT	Portugal	Chronic heart failure (non-diabetic HFrEF)	Dapagliflozin: 10 mg/d	Optimal Medical Therapy	40	6 months
Solomon et al. 2022 [67] (DELIVER Trial)	RCT	Multi-national (South America, Europe, Asia, North America)	Chronic heart failure	Dapagliflozin: 10 mg/d	Placebo	6236	27.6 months (IQR 20.4–33.6)
Von Lewinski et al. 2022 [70] (EMMY Trial)	RCT	Austria	Acute myocardial infarction	Empagliflozin: 10 mg/d	Placebo	476	6.0 months
Voors et al. 2022 [69] (EMPULSE Trial)	RCT	Multi-national (Europe, Asia, North America)	Acute heart failure	Empagliflozin: 10 mg/d	Placebo	530	3.0 months
Anker et al. 2021 [31] (EMPEROR-Preserved Trial)	RCT	Multi-national (South America, Europe, Asia, Africa, North America)	Chronic heart failure (HFpEF)	Empagliflozin: 10 mg/d	Placebo	5988	26.2 months (IQR 18.1–33.1)
Bhatt et al. 2021 [32] (SCORED Trial)	RCT	Multi-national (South America, Europe, Asia, North America)	Type 2 diabetes with chronic kidney disease	Sotagliflozin: 200–400 mg/d	Placebo	10,584	16.0 months (IQR 12.0–20.3)
Bhatt et al. 2021 [33] (SOLOIST-WHF Trial)	RCT	Multi-national (South America, Europe, Asia, North America)	Acute heart failure with diabetes	Sotagliflozin: 200–400 mg/d	Placebo	1222	9.0 months
Kosiborod et al. 2021 [15] (DARE-19 Trial)	RCT	Multi-national (South America, Europe, North America)	COVID-19 with cardiometabolic risk	Dapagliflozin: 10 mg/d	Placebo	1250	2.0 months
Lee et al. 2021 [51] (SUGAR-DM-HF Trial)	RCT	United Kingdom	Chronic heart failure (HFrEF with diabetes/prediabetes)	Empagliflozin: 10 mg/d	Placebo	105	8.3 months
Cannon et al. 2020 [35] (VERTIS-CV Trial)	RCT	(blank)	Type 2 diabetes with established atherosclerotic cardiovascular disease	Ertugliflozin: 5 or 15 mg/d	Placebo	8246	42.0 months
Damman et al. 2020 [37] (EMPA-RESPONSE-AHF Trial)	RCT	The Netherlands	Acute heart failure	Empagliflozin: 10 mg/d	Placebo	79	2.0 months
Heerspink et al. 2020 [39] (DAPA-CKD Trial)	RCT	Multi-national (South America, Europe, Asia, North America)	Chronic kidney disease (with or without diabetes)	Dapagliflozin: 10 mg/d	Placebo	4304	28.8 months (IQR 24.0–32.4)
Jensen et al. 2020 [45] (EMPIRE-HF Trial)	RCT	Denmark	Chronic heart failure (HFrEF)	Empagliflozin: 10 mg/d	Placebo	190	2.8 months
Katakami et al. 2020 [47] (UTOPIA Trial)	RCT	Japan	Type 2 diabetes	Tofogliflozin 20 mg/d	Conventional therapy	340	24 months
Packer et al. 2020 [60] (EMPEROR-Reduced Trial)	RCT	Multi-national (South America, Europe, Asia, North America)	Chronic heart failure (HFrEF)	Empagliflozin: 10 mg/d	Placebo	3730	16 months
Shimizu et al. 2020 [65] (EMBODY Trial)	RCT	Japan	Acute myocardial infarction with diabetes	Empagliflozin: 10 mg/d	Placebo	96	5.5 months
Kaku et al. 2019 [74]	RCT	Japan	Type 1 diabetes	Ipragliflozin 50 mg/d	Placebo	174	5.5 months
McMurray et al. 2019 [57] (DAPA-HF Trial)	RCT	Multi-national (South America, Europe, Asia, North America)	Chronic heart failure (HFrEF)	Dapagliflozin: 10 mg/d	Placebo	4744	18.2 months (range 0–27.8)
Nassif et al. 2019 [59] (DEFINE-HF Trial)	RCT	United States	Chronic heart failure (HFrEF)	Dapagliflozin: 10 mg/d	Placebo	263	2.8 months
Perkovic et al. 2019 [62] (CREDENCE Trial)	RCT	Multi-national (South America, Europe, Asia, North America)	Chronic kidney disease with type 2 diabetes	Canagliflozin 100 mg/d	Placebo	4401	31.4 months (range 0.24–54.4)
Wiviott et al. 2019 [71] (DECLARE–TIMI 58 Trial)	RCT	Multi-national (South America, Europe, Asia, North America)	Type 2 diabetes with cardiovascular risk	Dapagliflozin: 10 mg/d	Placebo	17,160	50.4 months (IQR 46.8–52.8)
Ikeda et al. 2015 [42]	RCT	Japan	Type 2 diabetes eligible patients treated with diet and exercise alone or with diet and exercise and a stable dose of metformin	Tofogliflozin: 2.5, 5, 10, 20 or 40 mg/d	Placebo	394	2.8 months
Kovacs et al. 2015 [49]	RCT	Multi-national (North America, Europe, Asia)	Type 2 diabetes who were receiving pioglitazone monotherapy or pioglitazone plus metformin	Empagliflozin: 10 or 25 mg/d	Placebo	498	5.5 months
Zinman et al. 2015 [73] (EMPA-REG OUTCOME Trial)	RCT	Multi-national (South America, Europe, Asia, North America)	Type 2 diabetes with established cardiovascular disease	Empagliflozin: 10 or 25 mg/d	Placebo	7020	37.2 months
Ji et al. 2014 [46]	RCT	China, Korea, Taiwan, India	Type 2 diabetes	Dapagliflozin 5 or 10 mg/d	Placebo	393	5.5 months
Inagaki et al. 2013 [43]	RCT	Japan	Type 2 diabetes	Canagliflozin 50, 100, 200 or 300 mg/d	Placebo	382	2.8 months

Abbreviations: RCT, randomized controlled trial; HF, heart failure; HFrEF, heart failure with reduced ejection fraction; HFpEF, heart failure with preserved ejection fraction; HFmrEF, heart failure with mildly reduced ejection fraction; CKD, chronic kidney disease; DM, diabetes mellitus; STEMI, ST-elevation myocardial infarction; ICU, intensive care unit; CPB, cardiopulmonary bypass; COVID-19, coronavirus disease 2019; IQR, interquartile range; mg/d, milligrams per day.

**Table 2:** Baseline characteristics of the included studies.

Study ID	Trial Name	Group	Age (Year)	Male n (%)	Weight (kg)	BMI (kg/m^2^)	Smoking
Berg et al. 2025 [75]	DAPA ACT HF-TIMI 68	Dapagliflozin 10 mg/day	69 [58,59,60,61,62,63,64,65,66,67,68,69,70,71,72,73,74,75,76,77]	815 (66.9%)		29.0 [24.9–34.7]	
		Placebo	68 [58,59,60,61,62,63,64,65,66,67,68,69,70,71,72,73,74,75,76]	771 (65.2%)		29.5 [25.4–35.4]	
Agarwal et al. 2025 [30]	CONFIDENCE	Empagliflozin 10 mg/day + Finerenone 10 or 20 mg/day	67.7 ± 10.0	202 (75.1)	83.0 ± 21.7	29.8 ± 6.7	
		Finerenone 10 or 20 mg/day	65.5 ± 10.7	203 (76.9)	81.9 ± 20.3	29.1 ± 5.7	
Heshmat et al. 2025 [40]		Dapagliflozin	52.1 ± 8.5	22 (81.5)	83.4 ± 12.9	28.3 ± 4.2	17 (63)
		Standard therapy	56 ± 6.4	21 (77.8)	80.7 ± 9.7	27.2 ± 3.5	14 (51.9)
Huang et al. 2025 [41]	DEFORM	Dapagliflozin 10 mg/day	61.98 ± 13.71	45 (86.5)		23.74 ± 2.44	
		Guideline-directed medical therapy	65.31 ± 13.87	38 (73.1)		24.21 ± 2.42	
Mocan et al. 2025 [58]		Dapagliflozin 10 mg/day + structured intravenous furosemide	63.63 ± 10.95	40 (81.6)	86.55 ± 20.35	Male: 28.49 ± 5.65 Female: 27.39 ± 5.08	
		Structured intravenous furosemide	65.31 ± 10.82	42 (85.7)	85.68 ± 25.33	Male: 29.12 ± 9.03 Female: 23.73 ± 2.67	
Raposeiras-Roubin et al. 2025 [63]	DapaTAVI	Dapagliflozin 10 mg/day	82.4 ± 5.6	306 (50.6)			
		Standard therapy	82.4 ± 5.5	313 (50.6)			
Snel et al. 2025 [66]		Empagliflozin 10 mg/day	69 ± 8	18 (72)		27.6 ± 3.4	
		Standard therapy	63 ± 10	22 (73)		28.2 ±5.1	
Zhou et al. 2025 [72]		Dapagliflozin 10 mg/day	65.27 ± 11.87	30 (60)			12 (24)
		Standard therapy	68.05 ± 9.79	28 (58.33)			16 (33.33)
Butler et al. 2024 [34]	EMPACT-MI	Empagliflozin 10 mg/day	63.6 ± 11.0	2448 (75.1)			
		Placebo	63.7 ± 10.8	2449 (75.1)			
James et al. 2024 [44]	DAPA MI	Dapagliflozin 10 mg/day	63.0 ± 11.06	1631 (80.8)	85.5 ± 15.87		
		Placebo	62.8 ± 10.64	1579 (79.0)	85.5 ± 16.54		
Kosiborod et al. 2024 [48]	The Accelerating COVID-19 Therapeutic Interventions and Vaccines 4 ACUTE (ACTIV-4a) trial	SGLT2i	72.6 ± 12.0	168 (58.5)		27.7 [23.8–33.0]	
		Standard therapy	71.0 ± 13.0	165 (57.3)		28.3 [23.7–34.5]	
Kumar et al. 2024 [50]		Empagliflozin 10 or 20 mg/day					
		Placebo					
Li et al. 2020 [52]		Janagliflozin 25 mg/day	49.0 ± 4.33	7 (78)	69.4 ± 5.95	25.3 ± 1.80	
		Janagliflozin 50 mg/day	48.0 ± 5.55	8 (89)	71.9 ± 9.36	26.1 ± 2.42	
		Dapagliflozin 10 mg/day	44.9 ± 6.68	7 (78)	72.4 ± 12.05	26.7 ± 2.65	
		Placebo	46.9 ± 4.28	7 (78)	68.8 ± 7.04	24.2 ± 2.54	
Liang et al. 2024 [53]		Dapagliflozin + sacubitril/valsartan	63 ± 12.2	18 (60)		25.9 ± 2.2	16 (53.3)
		Standard therapy + sacubitril/valsartan	64 ± 12.4	16 (53.3)		25.1 ± 2.7	13 (43.3)
Lin et al. 2024 [54]		Dapagliflozin 10 mg/day	62.3 ± 9.8	68 (68)			
		Placebo	63.1 ± 10.2	66 (66)			
McMurray et al. 2024 [56]	DETERMINE-preserved	Dapagliflozin 10 mg/day	73 [67,68,69,70,71,72,73,74,75,76,77,78]	162 (64)		29 [25,26,27,28,29,30,31,32,33,34]	
		Placebo	73 [73,74,75,76,77,78,79]	158 (62.9)		28 [25,26,27,28,29,30,31,32,33]	
McMurray et al. 2024 [56]	DETERMINE-reduced	Dapagliflozin 10 mg/day	69 [62,63,64,65,66,67,68,69,70,71,72,73,74,75,76]	111 (71.2)		28 [24,25,26,27,28,29,30,31,32,33,34]	
		Placebo	69 [60,61,62,63,64,65,66,67,68,69,70,71,72,73,74,75,76]	122 (77.7)		29 [24,25,26,27,28,29,30,31,32,33]	
Pastore et al. 2024 [61]	DAPA ECHO	Dapagliflozin 10 mg/day	68 ± 15	82 (36)		26.5 ± 3	14 (6)
		Optimal Medical Therapy	68 ± 12	84 (37)		28 ± 6	10 (4)
Tavares et al. 2024 [17]	DEFENDER	Dapagliflozin 10 mg/day	63.3 ± 14.9	139 (56)		25.1 [22.1–28.7]	46 (18.5)
		Standard therapy	64.5 ± 15.2	130 (50.2)		25.4 [22.1–29.3]	40 (15.4)
Emara et al. 2023 [38]	DAPA-RESPONSE-AHF	Dapagliflozin 10 mg/day	61.1 ± 11.8	35 (77.8)			21 (46.7)
		Placebo	63.9 ± 10	27 (64.3)			15 (35.7)
Liu et al. 2023 [55]		Empagliflozin 10 mg/day	67.42 ± 1.81	30 (55.56)		23.53 ± 2.14	
		Standard therapy	67.48 ± 1.85	31 (57.41)		24.03 ± 2.08	
RECOVERY Collaborative Group 2023 [16]	RECOVERY	Empagliflozin	61.1 (16.3)	1326 (63)			
		Usual care	61.8 (16.4)	1339 (62)			
The EMPA-KIDNEY Collaborative Group 2023 [68]	EMPA-KIDNEY trial	Empagliflozin 10 mg/day	63.9 ± 13.9	2207 (66.8)		29.7 ± 6.7	
		Placebo	63.8 ± 13.9	2210 (66.9)		29.8 ± 6.8	
Adel et al. 2022 [29]		Empagliflozin	55 [45.5–64]	27 (60)	75 [67.5–84.5]		
		Placebo	57 [50–66.75]	29 (60.4)	69.5 [65–83.75]		
Charaya et al. 2022 [36]		Dapagliflozin	72.6 ± 12.2	29 (58)			
		Standard therapy	74.2 ± 11.3	27 (52)			
Solomon et al. 2022 [67]	DELIVER	Dapagliflozin 10 mg/day	71.8 ± 9.6	1767 (56.4)			
		Placebo	71.5 ± 9.5	1749 (55.8)			
Reis et al. 2022 [64]		Dapagliflozin 10 mg/day + Optimal Medical Therapy	60.3 ± 11.6	17 (85)			17 (85)
		Optimal Medical Therapy	61.7 ± 14.8	16 (80)			11 (55)
Von Lewinski et al. 2022 [70]	EMMY	Empagliflozin 10 mg/day	57 [52,53,54,55,56,57,58,59,60,61,62,63,64]	195 (82)		27.7 [25.3–30.3]	171 (72)
		Placebo	57 [52,53,54,55,56,57,58,59,60,61,62,63,64,65]	197 (82)		27.2 [24.9–30.2]	170 (72)
Voors et al. 2022 [69]	EMPULSE	Empagliflozin 10 mg/day	71 [62,63,64,65,66,67,68,69,70,71,72,73,74,75,76,77,78]	179 (67.5)		28.35 [24.54–32.46]	
		Placebo	70 [59,60,61,62,63,64,65,66,67,68,69,70,71,72,73,74,75,76,77,78]	172 (64.9)		29.08 [24.69–33.60]	
Anker et al. 2021 [31]	EMPEROR-Preserved	Empagliflozin 10 mg/day	71.8 ± 9.3	1659 (55.4)		29.77 ± 5.8	
		Placebo	71.9 ± 9.6	1653 (55.3)		29.90 ± 5.9	
Bhatt et al. 2021 [32]	SCORED	Sotagliflozin 200–400 mg/day	69 [63,64,65,66,67,68,69,70,71,72,73,74]	2945 (55.7)		31.9 [28.1–36.2]	
		Placebo	69 [63,64,65,66,67,68,69,70,71,72,73,74]	2885 (54.5)		31.7 [28.0–36.1]	
Bhatt et al. 2021 [33]	SOLOIST-WHF	Sotagliflozin 200–400 mg/day	69 [63,64,65,66,67,68,69,70,71,72,73,74,75,76]	410 (67.4)		30.4 [26.3–34.3]	
		Placebo	70 [64,65,66,67,68,69,70,71,72,73,74,75,76]	400 (65.1)		31.1 [27.3–34.5]	
Kosiborod et al. 2021 [15]	DARE-19	Dapagliflozin 10 mg/day	61 ± 13.4	365 (58.4)		30.6 ± 6.2	29 (4.6)
		Placebo	61.8 ± 13.5	352 (56.3)		30.9 ± 6.4	20 (3.2)
Lee et al. 2021 [51]	SUGAR-DM-HF	Empagliflozin 10 mg/day	68.2 ± 11.7	34 (65.4)		30.9 ± 5.9	
		Placebo	69.2 ± 10.6	43 (81.1)		30.4 ± 5.1	
Cannon et al. 2020 [35]	VERTIS CV	Ertugliflozin 5 or 15 mg/day	64.4 ± 8.1	3866 (70.3)		31.9 ± 5.4	
		Placebo	64.4 ± 8	1903 (69.3)		32.0 ± 5.5	
Damman et al. 2020 [37]	EMPA-RESPONSE-AHF	Empagliflozin 10 mg/day	79 (73–83)	24 (60)	87 ± 23		
		Placebo	73 (61–83)	29 (74.4)	83 ± 20		
Heerspink et al. 2020 [39]	DAPA-CKD	Dapagliflozin 10 mg/day	61.8 ± 12.1	1443 (67.1)	81.5 ± 20.1	29.4 ± 6.0	283 (13.2)
		Placebo	61.9 ± 12.1	1436 (66.7)	82.0 ± 20.9	29.6 ± 6.3	301 (14.0)
Jensen et al. 2020 [45]	EMPIRE-HF	Empagliflozin	64 [57,58,59,60,61,62,63,64,65,66,67,68,69,70,71,72,73]	79 (83)		29 [27,28,29,30,31,32,33]	
		Placebo	63 [55,56,57,58,59,60,61,62,63,64,65,66,67,68,69,70,71,72]	83 (87)		29 [26,27,28,29,30,31,32,33]	
Katakami et al. 2020 [47]	UTOPIA	Tofogliflozin 20 mg/day	61.3 ± 9.3	99 (58.6)		27.0 ± 5.8	38 (22.6)
		Conventional therapy	60.9 ± 9.7	99 (58.2)		27.0 ± 4.6	29 (17.1)
Packer et al. 2020 [60]	EMPEROR-Reduced	Empagliflozin 10 mg/day	67.2 ± 10.8	1426 (76.5)		28.0 ± 5.5	
		Placebo	66.5 ± 11.2	1411 (75.6)		27.8 ± 5.3	
Shimizu et al. 2020 [65]	EMBODY	Empagliflozin 10 mg/day	63.9 ± 10.4	38 (82.6)	70.1 ± 13.7	25.2 ± 3.7	24 (52.2)
		Placebo	64.6 ± 11.6	39 (78)	68.1 ± 14.4	25.2 ± 4.1	27 (54.0)
Kaku et al. 2019 [74]		Ipragliflozin 50 mg/day	49.7 ± 13.1	54 (47.0)	66.06 ± 11.39	24.67 ± 2.95	
		Placebo	48.3 ± 12.8	27 (45.8)	64.68 ± 9.07	24.21 ± 2.82	
McMurray et al. 2019 [57]	DAPA-HF	Dapagliflozin 10 mg/day	66.2 ± 11.0	1809 (76.2)		28.2 ± 6.0	
		Placebo	66.5 ± 10.8	1826 (77)		28.1 ± 5.9	
Nassif et al. 2019 [59]	DEFINE-HF	Dapagliflozin 10 mg/day	62.2 ± 11.0	95 (72.5)		30.7 [27.3, 35.9]	
		Placebo	60.4 ± 12.0	98 (74.2)		30.6 [27.6, 36.4]	
Perkovic et al. 2019 [62]	CREDENCE	Canagliflozin 100 mg/day	62.9 ± 9.2	1440 (65.4)		31.4 ± 6.2	341 (15.5)
		Placebo	63.2 ± 9.2	1467 (66.7)		31.3 ± 6.2	298 (13.6)
Wiviott et al. 2019 [71]	DECLARE–TIMI 58	Dapagliflozin 10 mg/day	63.9 ± 6.8	5411 (63.1)		32.1 ± 6.0	
		Placebo	64.0 ± 6.8	5327 (62.1)		32.0 ± 6.1	
Ikeda et al. 2015 [42]		Tofogliflozin 2.5 mg/day	53.3 ± 10.86	34 (51.5)	85.23 ± 16.465	31.33 ± 4.878	
		Tofogliflozin 5 mg/day	54.8 ± 10.53	31 (47.7)	82.15 ± 16.346	30.56 ± 5.230	
		Tofogliflozin 10 mg/day	54.5 ± 10.70	34 (51.5)	83.41 ± 16.563	30.4 ± 4.910	
		Tofogliflozin 20 mg/day	56.3 ± 9.79	43 (67.2)	84.91 ± 17.305	30.09 ± 4.652	
		Tofogliflozin 40 mg/day	57.5 ± 9.31	31 (46.3)	81.68 ± 18.692	30.36 ± 4.892	
		Placebo	53.9 ± 11.12	36 (54.5)	83.73 ± 19.201	30.37 ± 5.466	
Kovacs et al. 2015 [49]		Empagliflozin 10 mg/day	54.7 ± 9.9	85 (50.6)	78.0 ± 19.2	29.2 ± 5.6	
		Empagliflozin 25 mg/day	54.2 ± 8.9	83 (50.3)	78.9 ± 19.9	29.1 ± 5.5	
		Placebo	54.6 ± 10.5	73 (44.2)	78.1 ± 20.1	29.3 ± 5.4	
Zinman et al. 2015 [73]	EMPA-REG OUTCOME	Empagliflozin 10 mg/day	63.0 ± 8.6)	1653 (70.5)	85.9 ± 18.8	30.6 ± 5.2	
		Empagliflozin 25 mg/day	63.2 ± 8.6	1683 (71.9)	86.5 ± 19	30.6 ± 5.3	
		Empagliflozin 10 or 25 mg/day	63.1 ± 8.6	3336 (71.2)	86.2 ± 18.9	30.6 ± 5.3	
		Placebo	63.2 ± 8.8	1680 (72.0)	86.6 ± 19.1	30.7 ± 5.2	
Ji et al. 2014 [46]		Dapagliflozin 5 mg/day	53.0 ± 11.07	84 (65.6)	68.89 ± 11.43	25.17 ± 3.29	
		Dapagliflozin 10 mg/day	51.2 ± 9.89	86 (64.7)	70.92 ± 11.64	25.76 ± 3.43	
		Placebo	49.9 ± 10.87	87 (65.9)	72.18 ± 13.23	25.93 ± 3.64	
Inagaki et al. 2013 [43]		Canagliflozin 50 mg/day	57.4 ± 10.8	50 (61.0)	65.77 ± 13.56	26.41 ± 4.34	
		Canagliflozin 100 mg/day	57.7 ± 10.5	52 (70.3)	68.61 ± 14.86	25.11 ± 4.13	
		Canagliflozin 200 mg/day	57.0 ± 10.7	49 (64.5)	68.97 ± 14.50	25.61 ± 4.64	
		Canagliflozin 300 mg/day	57.1 ± 10.1	55 (73.3)	71.30 ± 12.19	25.51 ± 4.30	
		Placebo	57.7 ± 11.0	54 (72.0)	72.56 ± 15.36	25.89 ± 3.68	

BMI, body mass index; IQR, interquartile range; kg, kilogram; m^2^, square meter; mg/day, milligrams per day. Data are presented as mean ± SD, median [IQR], or n (%), as reported.

## Data Availability

No new data were created or analyzed in this study. Data sharing is not applicable to this article.

## Supplementary Materials

The following supporting information can be downloaded at: https://www.mdpi.com/article/10.3390/ijms27073168/s1.
