# Supplementary material for: Impact of SGLT2 Inhibitors on Mortality Across Different Populations: A Systematic Review and Meta-Analysis
Source: Int J Mol Sci. 2026 Mar 31;27(7):3168. doi: 10.3390/ijms27073168 (PMC13073246; doi:10.3390/ijms27073168)
Supplement: Supplementary file 1 [file ijms-27-03168-s001.zip › ijms-4167324-supplementaryfileS1.pdf]

Full Search strategy	
#1	(SGLT2 OR SGLT-2 OR "sodium glucose transport" OR "sodium-glucose transport" OR "sodium glucose cotransporter" OR "sodium-glucose cotransporter" OR "sodium glucose co-transporter" OR "sodium-glucose co-transporter" OR Empagliflozin OR Dapagliflozin OR Canagliflozin OR Ertugliflozin OR Sotagliflozin OR Ipragliflozin OR Luseogliflozin OR Tofogliflozin OR Bexagliflozin OR Licogliflozin)
#2	(Mortality OR mortalities OR death* OR Fatality)
#3	#1 AND #2
**PubMed/Medline**	
(SGLT2[Title/Abstract] OR SGLT-2[Title/Abstract] OR "Sodium-Glucose Transport Proteins"[Mesh] OR "sodium glucose transport"[Title/Abstract] OR "sodium-glucose transport"[Title/Abstract] OR "sodium glucose cotransporter"[Title/Abstract] OR "sodium-glucose cotransporter"[Title/Abstract] OR "sodium glucose co-transporter"[Title/Abstract] OR "sodium-glucose co-transporter"[Title/Abstract] OR Empagliflozin[Title/Abstract] OR Dapagliflozin[Title/Abstract] OR Canagliflozin[Title/Abstract] OR Ertugliflozin[Title/Abstract] OR Sotagliflozin[Title/Abstract] OR Ipragliflozin[Title/Abstract] OR Luseogliflozin[Title/Abstract] OR Tofogliflozin[Title/Abstract] OR Bexagliflozin[Title/Abstract] OR Licogliflozin[Title/Abstract]) AND (Mortality[Title/Abstract] OR mortalities[Title/Abstract] OR death*[Title/Abstract] OR Fatality[Title/Abstract])<br><br>Search Date: March 12, 2025<br><br>Results: 4,733	
Scopus	
TITLE-ABS-KEY ((SGLT2 OR SGLT-2 OR "sodium glucose transport" OR "sodium-glucose transport" OR "sodium glucose cotransporter" OR "sodium-glucose cotransporter" OR "sodium glucose co-transporter" OR "sodium-glucose co-transporter" OR Empagliflozin OR Dapagliflozin OR Canagliflozin OR Ertugliflozin OR Sotagliflozin OR Ipragliflozin OR Luseogliflozin OR Tofogliflozin OR Bexagliflozin OR Licogliflozin) AND (Mortality OR mortalities OR death* OR Fatality))<br><br>Search Date: March 12, 2025<br><br>Results: 11,591	
Web of Science	
TS= ((SGLT2 OR SGLT-2 OR "sodium glucose transport" OR "sodium-glucose transport" OR "sodium glucose cotransporter" OR "sodium-glucose cotransporter" OR "sodium glucose co-transporter" OR "sodium-glucose co-transporter" OR Empagliflozin OR Dapagliflozin OR Canagliflozin OR Ertugliflozin OR Sotagliflozin OR Ipragliflozin OR Luseogliflozin OR Tofogliflozin OR Bexagliflozin OR Licogliflozin) AND (Mortality OR mortalities OR death* OR Fatality))<br><br>Search Date: March 12, 2025<br><br>Results: 6,287	
Cochrane CENTRAL	

Title Abstract Keyword ((SGLT2 OR SGLT-2 OR "sodium glucose transport" OR "sodium-glucose transport" OR "sodium glucose cotransporter" OR "sodium-glucose cotransporter" OR "sodium glucose co-transporter" OR "sodium-glucose co-transporter" OR Empagliflozin OR Dapagliflozin OR Canagliflozin OR Ertugliflozin OR Sotagliflozin OR Ipragliflozin OR Luseogliflozin OR Tofogliflozin OR Bexagliflozin OR Licogliflozin) AND (Mortality OR mortalities OR death* OR Fatality))

Search Date: March 12, 2025

Results: 1,845

Table S1. Detailed Search Strategy.

Study ID	Trial Name	Group	Comorbidities
Agarwal et al. 2025 [30]	CONFIDENCE	Empagliflozin 10 mg/day + Finerenone 10 or 20 mg/day	History of atherosclerotic cardiovascular disease, 75 (27.9)
		Finerenone 10 or 20 mg/day	History of atherosclerotic cardiovascular disease, 71 (26.9)
Heshmat et al. 2025 [40]		Dapagliflozin	Hypertension, 14 (51.9)<br>Dyslipidemia, 12 (44.4)<br>Previous myocardial infarction, 3 (11.1)
		Standard therapy	Hypertension, 14 (51.9)<br>Dyslipidemia, 8 (29.6)<br>Previous myocardial infarction, 3 (11.1)
Huang et al. 2025 [41]	DEFORM	Dapagliflozin 10 mg/day	Coronary heart disease, 28 (53.8)<br>Hypertension, 21 (41.2)<br>Diabetes mellitus, 20 (38.5)<br>Atrial fibrillation, 14 (26.9)
		Guideline-directed medical therapy	Coronary heart disease, 29 (55.8)<br>Hypertension, 19 (36.5)<br>Diabetes mellitus, 17 (32.7)<br>Atrial fibrillation, 19 (36.5)
Mocan et al. 2025 [58]		Dapagliflozin 10 mg/day + structured intravenous furosemide	Atrial fibrillation, 16 (32.7)<br>T2DM, 10 (20.4)<br>Ischenic heart disease, 14 (28.6)<br>Chronic kideny disease, 5 (10.2)<br>Severe mitral regurgitation, 9 (18.4)<br>Severe tricuspid regurgitation, 8 (16.3)
		Structured intravenous furosemide	Atrial fibrillation, 20 (40.8)<br>T2DM, 15 (30.6)<br>Ischenic heart disease, 10 (20.4)<br>Chronic kideny disease, 13 (26.5)<br>Severe mitral regurgitation, 15 (30.6)<br>Severe tricuspid regurgitation, 14 (28.6)
Raposeiras-Roubin et al. 2025 [63]	DapaTAVI	Dapagliflozin 10 mg/day	Hypertension, 518 (85.6)<br>Diabetes mellitus type 2, 264 (43.6)<br>Atrial fibrillation, 250 (41.3)<br>Coronary artery disease, 237 (39.2)<br>Previous stroke, 61 (10.1)

			Previous myocardial infarction, 51 (8.4)<br>Peripheral-artery disease, 51 (8.4)<br>Hypertension, 519 (84.0)<br>Diabetes mellitus type 2, 273 (44.2)
		Standard therapy	Atrial fibrillation, 274 (44.3)<br>Coronary artery disease, 197 (31.9)<br>Previous stroke, 69 (11.2)<br>Previous myocardial infarction, 52 (8.4)<br>Peripheral-artery disease, 43 (7.0)
Snel et al. 2025 [66]		Empagliflozin 10 mg/day	T2DM, n=3 (12)
		Standard therapy	T2DM, n=2 (7)
Zhou et al. 2025 [72]		Dapagliflozin 10 mg/day	Hypertension, 37 (74)<br>Diabetes mellitus, 25 (50)<br>Stroke, 14 (28)<br>Hyperlipidemia, 8 (16)
		Standard therapy	Hypertension, 38 (79.17)<br>Diabetes mellitus, 30 (62.5)<br>Stroke, 18 (37.5)<br>Hyperlipidemia, 6 (12.5)
Butler et al. 2024 [34]	EMPACT-MI	Empagliflozin 10 mg/day	Hypertension, 2262 (69.4)<br>Diabetes mellitus type 2, 1035 (31.7)<br>Previous myocardial infarction, 388 (11.9)<br>Atrial fibrillation, 358 (11.0)<br>Peripheral artery disease, 172 (5.3)
		Placebo	Hypertension, 2276 (69.8)<br>Diabetes mellitus type 2, 1046 (32.1)<br>Previous myocardial infarction, 459 (14.1)<br>Atrial fibrillation, 361 (11.1)<br>Peripheral artery disease, 180 (5.5)
James et al. 2024 [44]	DAPA MI	Dapagliflozin 10 mg/day	History of MI, 178 (8.8)<br>History of Stroke, 46 (2.3)
		Placebo	History of MI, 189 (9.5)<br>History of Stroke, 50 (2.5)
Kosiborod et al. 2024 [48]	The Accelerating COVID-19 Therapeutic Interventions and Vaccines 4 ACUTE (ACTIV-4a) trial	SGLTi	Hypertension, 198 (69.0)<br>T2DM, 112/286 (39.2)<br>Coronary artery disease, 66 (23.0)<br>Heart failure, 64 (22.3)<br>Cerebrovascular disease, 46 (16.0)<br>Chronic kidney disease, 45/286 (15.7)<br>COPD, 30/282 (10.6)<br>Asthma, 18/282 (6.4)<br>Peripheral arterial disease, 17 (5.9)<br>Liver disease, 6/286 (2.1)
		Standard therapy	Hypertension, 193 (67.0)<br>T2DM, 110/286 (38.5)<br>Coronary artery disease, 71 (24.7)<br>Heart failure, 53 (18.4)<br>Cerebrovascular disease, 32 (11.1)<br>Chronic kidney disease, 46/286 (16.1)<br>COPD, 33/283 (11.7)

			Asthma, 30/283 (10.6)<br>Peripheral arterial disease, 15 (5.2)<br>Liver disease, 11/286 (3.8)
Kumar et al. 2024 [50]		Empagliflozin 10 or 20 mg<br>Placebo	
Li et al. 2024 [52]		Janagliflozin 25 mg<br>Janagliflozin 50 mg<br>Dapagliflozin 10 mg<br>Placebo	
Liang et al. 2024 [53]		Dapagliflozin + sacubitril/valsartan	Hypertension, 22 (73.3)<br>Diabetes, 11 (36.7)<br>Cerebral infarction, 6 (20.0)
		Standard therapy + sacubitril/valsartan	Hypertension, 24 (80.0)<br>Diabetes, 13 (43.3)<br>Cerebral infarction, 5 (16.7)
Lin et al. 2024 [54]		Dapagliflozin 10 mg/day	Hypertension, 76 (76)<br>Coronary artery disease, 54 (54)<br>Diabetes, 42 (42)<br>Renal impairment, 28 (28)
		Placebo	Hypertension, 78 (78)<br>Coronary artery disease, 56 (56)<br>Diabetes, 40 (40)<br>Renal impairment, 30 (30)
McMurray et al. 2024 [56]	DETERMINE -preserved	Dapagliflozin 10 mg/day	T2DM, 109 (43.1)<br>Atrial fibrillation, 137 (54.2)
		Placebo	T2DM, 111 (44.2<br>Atrial fibrillation, 125 (49.8)
McMurray et al. 2024 [56]	DETERMINE -reduced	Dapagliflozin 10 mg/day	T2DM, 72 (46.2)<br>Atrial fibrillation, 55 (35.3)
		Placebo	T2DM, 73 (46.5)<br>Atrial fibrillation, 62 (39.5
Pastore et al. 2024 [61]	DAPA ECHO	Dapagliflozin 10 mg/day	Dyslipidemia, 83 (35)<br>Hypertension, 72 (31)<br>Coronary artery disease, 30 (13)<br>Paroxysmal atrial fibrillation, 18 (8)
		Optimal Medical Therapy	Dyslipidemia, 75 (33)<br>Hypertension, 70 (30)<br>Coronary artery disease, 41 (18)<br>Paroxysmal atrial fibrillation, 16 (7)
Tavares et al. 2024 [17]	DEFENDER	Dapagliflozin 10 mg/day	Hypertension, 152 (61.3)<br>Type 2 diabetes, 77 (31.0)<br>Dyslipidemia, 56 (22.6)<br>Heart failure, 47 (19.0)<br>Prior myocardial infarction, 38 (15.3)<br>Localized cancer, 34 (13.7)<br>Chronic kidney disease, 29 (11.7)<br>Prior stroke, 19 (7.7)<br>Metastatic cancer, 13 (5.2)<br>Hematological cancer, 4 (1.6)

			HIV infection, 2 (0.8)<br>Solid organ transplant, 2 (0.8)
		Standard therapy	Hypertension, 179 (69.1)<br>Type 2 diabetes, 91 (35.1)<br>Dyslipidemia, 71 (27.4)<br>Heart failure, 40 (15.4)<br>Prior myocardial infarction, 28 (10.8)<br>Localized cancer, 35 (13.5)<br>Chronic kidney disease, 24 (9.3)<br>Prior stroke, 30 (11.6)<br>Metastatic cancer, 13 (5.0)<br>Hematological cancer, 4 (1.5)<br>HIV infection, 3 (1.2)<br>Solid organ transplant, 1 (0.4)
Emara et al. 2023 [38]	DAPA-RESPONSE-AHF	Dapagliflozin 10 mg/day	Hypertension, 22 (48.9)<br>T2DM, 16 (35.6)<br>Atrial fibrillation, 14 (31.1)<br>Dyslipidemia, 12 (26.7)<br>COPD, 8 (17.8)<br>Ischemic etiology, 8 (17.8)
		Placebo	Hypertension, 21 (50)<br>T2DM, 22 (52.4)<br>Atrial fibrillation, 13 (31)<br>Dyslipidemia, 15 (35.7)<br>COPD, 2 (4.8)<br>Ischemic etiology, 12 (28.6)
Liu et al. 2023 [55]		Empagliflozin 10 mg/day	
		Standard therapy	
RECOVERY Collaborative Group 2023 [16]	RECOVERY	Empagliflozin	Chronic lung disease, 533 (25)<br>Heart disease, 471 (22)<br>Diabetes, 333 (16)<br>Severe kidney impairment, 66 (3)<br>HIV, 21 (1)<br>Severe liver disease, 20 (1)<br>Tuberculosis, 9 (<1)
		Usual care	Chronic lung disease, 508 (24)<br>Heart disease, 455 (21)<br>Diabetes, 356 (16)<br>Severe kidney impairment, 80 (4)<br>Severe liver disease, 21 (1)<br>HIV, 13 (1)<br>Tuberculosis, 7 (<1)
The EMPA-KIDNEY Collaborative Group 2023 [68]	EMPA-KIDNEY trial	Empagliflozin 10 mg/d	History of diabetes, 1525 (46.2)<br>T2DM, 1470 (96.4)<br>T1DM, 34 (2.2)<br>History of cardiovascular disease, 861 (26.1)<br>GFR <30 ml/min/1.73 m2, 1131 (34.2)<br>Albumin-to-creatinine ratio >300, 1712 (51.8)
		Placebo	History of diabetes, 1515 (45.8)<br>T2DM, 1466 (96.8)

			T1DM, 34 (2.2)<br>History of cardiovascular disease, 904 (27.4)<br>GFR <30 ml/min/1.73 m2, 1151 (34.8)<br>Albumin-to-creatinine ratio >300, 1705 (51.6)
Adel et al. 2022 [29]		Empagliflozin	Chronic kideny disease, 4 (8.9)<br>Hypertension, 26 (57.8)<br>Cerebrovascular accident, 1 (2.2)
		Placebo	Chronic kideny disease, 3 (6.3)<br>Hypertension, 32 (66.7)<br>Cerebrovascular accident, 2 (4.2)
Charaya et al. 2022 [36]		Dapagliflozin	De novo acute HF, 17 (34)<br>Myocardial infarction, 26 (52)<br>T2DM, 15 (30)<br>Arterial hypertension, 46 (92)<br>Atrial fibrillation, 25 (50)<br>Chronic kideny disease, 26 (52)<br>Anaemia, 20 (40)<br>Stroke/TIA, 7 (14)<br>COPD, 16 (32)<br>Bronchial asthma, 8 (15)<br>Peptic ulcer disease, 7 (13)
		Standard therapy	De novo acute HF, 20 (38)<br>Myocardial infarction, 25 (48)<br>T2DM, 16 (30)<br>Arterial hypertension, 48 (92)<br>Atrial fibrillation, 30 (57)<br>Chronic kideny disease, 31 (59)<br>Anaemia, 22 (42)<br>Stroke/TIA, 5 (10)<br>COPD, 20 (38)<br>Bronchial asthma, 9 (17)<br>Peptic ulcer disease, 5 (9)
Solomon et al. 2022 [67]	DELIVER	Dapagliflozin 10 mg/day	T2DM, 1401 (44.7)<br>Hypertension, 2755 (88)<br>Previous left ventricular ejection fraction ≤40%, 572 (18.3)
		Placebo	T2DM, 1405 (44.9)<br>Hypertension, 2798 (89.3)<br>Previous left ventricular ejection fraction ≤40%, 579 (18.5)
Reis et al. 2022 [64]		Dapagliflozin 10 mg/day + Optimal Medical Therapy	Hypertension, 14 (70)<br>Dyslipidemia, 16 (80)<br>Atrial fibrillation, 6 (30)<br>Chronic kidney disease, 5 (25)<br>Peripheral artery disease, 4 (20)<br>COPD, 5 (25)
		Optimal Medical Therapy	Hypertension, 11 (55)<br>Dyslipidemia, 10 (50)<br>Atrial fibrillation, 6 (30)<br>Chronic kidney disease, 3 (15)<br>Peripheral artery disease, 5 (25)<br>COPD, 6 (30)

Von Lewinski et al. 2022 [70]	EMMY	Empagliflozin 10 mg/day	Hypertension, 92 (39)<br>Dyslipidaemia, 71 (30)<br>Obesity, 68 (29)<br>Type 2 diabetes, 30 (13)<br>Coronary artery disease, 28 (12)<br>History of myocardial infarction, 14 (5.9)<br>Depression, 15 (6.3)<br>History of carcinoma, 11 (4.6)<br>History of stroke, 5 (2.1)
		Placebo	Hypertension, 107 (45)<br>Dyslipidaemia, 64 (27)<br>Obesity, 70 (29)<br>Type 2 diabetes, 33 (14)<br>Coronary artery disease, 25 (10)<br>History of myocardial infarction, 9 (3.8)<br>Depression, 9 (3.8)<br>History of carcinoma, 13 (5.4)<br>History of stroke, 1 (0.4)
Voors et al. 2022 [69]	EMPULSE	Empagliflozin 10 mg/d	Hypertension, 205 (77.4)<br>Valvular heart disease, 173 (65.3)<br>Atrial fibrillation, 134 (50.6)<br>Diabetes, 124 (46.8)<br>Myocardial infarction, 66 (24.9)
		Placebo	Hypertension, 221 (83.4)<br>Valvular heart disease, 167 (63.0)<br>Atrial fibrillation, 128 (48.3)<br>Diabetes, 116 (43.8)<br>Myocardial infarction, 62 (23.4)
Anker et al. 2021 [31]	EMPEROR-Preserved	Empagliflozin 10 mg/d	schemic heart failure, 1079 (36)<br>Nonischemic heart failure, 1917 (64)<br>Hospitalization for heart failure, 699 (23.3)<br>Atrial fibrillation, 1543 (51.5)<br>Diabetes mellitus, 1466 (48.9)<br>Hypertension, 2721 (90.8)<br>eGFR <60 ml/min/1.73 m2, 1504 (50.2)
		Placebo	Ischemic heart failure, 1038 (34.7)<br>Nonischemic heart failure, 1953 (65.3)<br>Hospitalization for heart failure, 670 (22.4)<br>Atrial fibrillation, 1514 (50.6)<br>Diabetes mellitus, 1472 (49.2)<br>Hypertension, 2703 (90.4)<br>eGFR <60 ml/min/1.73 m2, 1484 (49.6)
Bhatt et al. 2021 [32]	SCORED	Sotagliflozin 200-400 mg/day	History of heart failure, 1640 (31)<br>Previous myocardial infarction, 1051 (19.9)<br>Previous coronary revascularization, 1208 (22.8)<br>Previous stroke, 472 (8.9)
		Placebo	History of heart failure, 1643 (31)<br>Previous myocardial infarction, 1057 (20)<br>Previous coronary revascularization, 1167 (22.1)<br>Previous stroke, 474 (9)

Bhatt et al. 2021 [33]	SOLOIST-WHF	Sotagliflozin 200-400 mg/d<br>Placebo	
Kosiborod et al. 2021 [15]	DARE-19	Dapagliflozin 10 mg/day	Type 2 diabetes, 312 (49.9)<br>Heart failure, 44 (7.0)<br>Hypertension, 526 (84.2)<br>Atherosclerotic cardiovascular disease, 93 (14.9)<br>Chronic kidney disease, 38 (6.1)<br>Chronic obstructive pulmonary disease, 25 (4.0)
		Placebo	Type 2 diabetes, 324 (51.8)<br>Heart failure, 46 (7.4)<br>Hypertension, 534 (85.4)<br>Atherosclerotic cardiovascular disease, 106 (17.0)<br>Chronic kidney disease, 44 (7.0)<br>Chronic obstructive pulmonary disease, 32 (5.1)
Lee et al. 2021 [51]	SUGAR-DM-HF	Empagliflozin 10 mg/d	Type 2 diabetes, 40 (76.9)<br>Prediabetes, 12 (23.1)<br>Hypertension, 39 (75.0)<br>Coronary artery disease, 36 (69.2)<br>Prior myocardial infarction, 27 (75.0)<br>Prior heart failure hospitalization, 21 (40.4)<br>Stroke, 3 (5.8)
		Placebo	Type 2 diabetes, 42 (79.2)<br>Prediabetes, 11 (20.8)<br>Hypertension, 35 (66.0)<br>Coronary artery disease, 38 (71.7)<br>Prior myocardial infarction, 35 (92.1)<br>Prior heart failure hospitalization, 31 (58.5)<br>Stroke, 8 (15.1)
Cannon et al. 2020 [35]	VERTIS CV	Ertugliflozin 5 or 15 mg/d	Coronary artery disease, 4144 (75.4)<br>Myocardial infarction, 2625 (47.7)<br>Heart failure, 1286 (23.4)<br>Cerebrovascular disease, 1276 (23.2)<br>Stroke, 1181 (21.5)<br>Peripheral arterial disease, 1029 (18.7)<br>eGFR <60 ml/min/1.73 m2, 1199 (21.8)
		Placebo	Coronary artery disease, 2112 (76.9)<br>Myocardial infarction, 1329 (48.4)<br>Heart failure, 672 (24.5)<br>Cerebrovascular disease, 613 (22.3)<br>Stroke, 558 (20.3)<br>Peripheral arterial disease, 512 (18.6)<br>eGFR <60 ml/min/1.73 m2, 608 (22.1)
Damman et al. 2020 [37]	EMPA-RESPONSE-AHF	Empagliflozin 10 mg/day	Atrial fibrillation/flutter, 31 (78)<br>Hypertension, 27 (68)<br>Diabetes mellitus type 2, 15 (38)<br>Cancer, 15 (38)<br>Myocardial infarction, 12 (30)<br>COPD, 11 (28)<br>Ischaemic aetiology, 11 (28)<br>Cerebrovascular accident, 2 (5)

		Placebo	Atrial fibrillation/flutter, 25 (64)<br>Hypertension, 22 (56)<br>Diabetes mellitus type 2, 11 (28)<br>Cancer, 5 (13)<br>Myocardial infarction, 15 (38)<br>COPD, 10 (26)<br>Ischaemic aetiology, 11 (29)<br>Cerebrovascular accident, 2 (5)
Heerspink et al. 2020 [39]	DAPA-CKD	Dapagliflozin 10 mg/day	T2DM, 1455 (67.6)<br>Cardiovascular disease, 813 (37.8)<br>Heart failure, 235 (10.9)
		Placebo	T2DM, 1451 (67.4)<br>Cardiovascular disease, 797 (37.0)<br>Heart failure, 233 (10.8)
Jensen et al. 2020 [45]	EMPIRE-HF	Empagliflozin	Ischemic heart disease, 50 (53)<br>Atrial fibrillation or flutter, 36 (38)<br>T2DM (History/Newly Diagnosed), 19 (20)<br>Chronic kidney disease (Stage 3), 11 (12)
		Placebo	Ischemic heart disease, 53 (56)<br>Atrial fibrillation or flutter, 34 (36)<br>T2DM (History/Newly Diagnosed), 14 (15)<br>Chronic kidney disease (Stage 3), 12 (13)
Katakami et al. 2020 [47]	UTOPIA	Tofogliflozin 20 mg/d	Hypertension, 88 (52.1)<br>Dyslipidemia, 107 (63.3)<br>Diabetic retinopathy, 28 (16.8)<br>Diabetic nephropathy, 48 (28.4)
		Conventional therapy	Hypertension, 105 (61.8)<br>Dyslipidemia, 122 (71.8)<br>Diabetic retinopathy, 33 (19.5)<br>Diabetic nephropathy, 53 (31.2)
Packer et al. 2020 [60]	EMPEROR-Reduced	Empagliflozin 10 mg/d	Ischemic heart failure, 983 (52.8)<br>Nonischemic heart failure, 880 (47.2)<br>Hospitalization for heart failure, 577 (31.0)<br>Atrial fibrillation, 664 (35.6)<br>Diabetes mellitus, 927 (49.8)<br>Hypertension, 1349 (72.4)<br>eGFR <60 ml/min/1.73 m2, 893 (48.0)
		Placebo	Ischemic heart failure, 946 (50.7)<br>Nonischemic heart failure, 921 (49.3)<br>Hospitalization for heart failure, 574 (30.7)<br>Atrial fibrillation, 705 (37.8)<br>Diabetes mellitus, 929 (49.8)<br>Hypertension, 1349 (72.3)<br>eGFR <60 ml/min/1.73 m2, 906 (48.6)
Shimizu et al. 2020 [65]	EMBODY	Empagliflozin 10 mg/day	Hypertension, 38 (82.6)<br>Dyslipidemia, 34 (73.9)<br>Cerebrocardiovascular disease, 7 (15.2)
		Placebo	Hypertension, 39 (78.0)<br>Dyslipidemia, 36 (72.0)<br>Cerebrocardiovascular disease, 11 (22.0)

Kaku et al. 2019 [74]		Ipragliflozin 50 mg/d	eGFR 30 to < 60 mL/min/1.73 m^2, 3 (2.6)<br>HbA1c ≥ 63 mmol/mol, 94 (81.7)
		Placebo	eGFR 30 to < 60 mL/min/1.73 m^2, 1 (1.7)<br>HbA1c ≥ 63 mmol/mol, 47 (79.7)
McMurray et al. 2019 [57]	DAPA-HF	Dapagliflozin 10 mg/day	Ischemic heart failure, 1316 (55.5)<br>Nonischemic heart failure, 857 (36.1)<br>Hospitalization for heart failure, 1124 (47.4)<br>Atrial fibrillation, 916 (38.6)<br>Diabetes mellitus, 993 (41.8)<br>GFR <60 ml/min/1.73 m2, 962 (40.6)
		Placebo	Ischemic heart failure, 1358 (57.3)<br>Nonischemic heart failure, 830 (35.0)<br>Hospitalization for heart failure, 1127 (47.5)<br>Atrial fibrillation, 902 (38.0)<br>Diabetes mellitus, 990 (41.8)<br>GFR <60 ml/min/1.73 m2, 964 (40.7)
Nassif et al. 2019 [59]	DEFINE-HF	Dapagliflozin 10 mg/day	T2DM, 81 (61.8)<br>Ischemic heart disease, 70 (53.4)<br>Atrial Fibrillation, 57 (43.5)<br>Previous hospitalization for heart failure, 101 (77.1)
		Placebo	T2DM, 85 (64.4)<br>Ischemic heart disease, 69 (52.3)<br>Atrial Fibrillation, 49 (37.1)<br>Previous hospitalization for heart failure, 108 (81.8)
Perkovic et al. 2019 [62]	CREDENCE	Canagliflozin 100 mg/day	Hypertension, 2131 (96.8)<br>Heart failure, 329 (14.9)<br>Cardiovascular disease, 1113 (50.5)<br>Amputation, 119 (5.4)
		Placebo	Hypertension, 2129 (96.8)<br>Heart failure, 323 (14.7)<br>Cardiovascular disease, 1107 (50.3)<br>Amputation, 115 (5.2)
Wiviott et al. 2019 [71]	DECLARE–TIMI 58	Dapagliflozin 10 mg/day	Established atherosclerotic cardiovascular disease, 3474 (40.5)<br>History of coronary artery disease, 2824 (32.9)<br>History of peripheral artery disease, 522 (6.1)<br>History of cerebrovascular disease, 653 (7.6)<br>History of heart failure, 852 (9.9)
		Placebo	Established atherosclerotic cardiovascular disease, 3500 (40.8)<br>History of coronary artery disease, 2834 (33)<br>History of peripheral artery disease, 503 (5.9)<br>History of cerebrovascular disease, 648 (7.6)<br>History of heart failure, 872 (10.2)
Ikeda et al. 2015 [42]		Tofogliflozin 2.5 mg<br>Tofogliflozin 5 mg<br>Tofogliflozin 10 mg<br>Tofogliflozin 20 mg<br>Tofogliflozin 40 mg<br>Placebo<br>Empagliflozin 10 mg	

Kovacs et al. 2015 [49]		Empagliflozin 25 mg<br>Placebo	
Zinman et al. 2015 [73]	EMPA-REG OUTCOME	Empagliflozin 10 mg/d	Coronary artery disease, 1782 (76.0)<br>Multi-vessel coronary artery disease, 1078 (46.0)<br>History of myocardial infarction, 1107 (47.2)<br>History of stroke, 535 (22.8)<br>Peripheral artery disease, 465 (19.8)<br>Single vessel coronary artery disease, 258 (11.0)<br>Cardiac failure, 240 (10.2)<br>eGFR 60 to <90 mL/min/1.73m2, 1221 (52.1)<br>eGFR <60 mL/min/1.73m2, 605 (25.8)<br>Urine albumin-to-creatinine ratio 30 to 300 mg/g, 645 (27.5)<br>Urine albumin-to-creatinine ratio >300 mg/g, 261 (11.1)
		Empagliflozin 25 mg/d	Coronary artery disease, 1763 (75.3)<br>Multi-vessel coronary artery disease, 1101 (47.0)<br>History of myocardial infarction, 1083 (46.2)<br>History of stroke, 549 (23.4)<br>Peripheral artery disease, 517 (22.1)<br>Single vessel coronary artery disease, 240 (10.2)<br>Cardiac failure, 222 (9.5)<br>eGFR 60 to <90 mL/min/1.73m2, 1202 (51.3)<br>eGFR <60 mL/min/1.73m2, 607 (25.9)<br>Urine albumin-to-creatinine ratio 30 to 300 mg/g, 693 (29.6)<br>Urine albumin-to-creatinine ratio >300 mg/g, 248 (10.6)
		Empagliflozin 10 or 25 mg/d	Coronary artery disease, 3545 (75.6)<br>Multi-vessel coronary artery disease, 2179 (46.5)<br>History of myocardial infarction, 2190 (46.7)<br>History of stroke, 1084 (23.1)<br>Peripheral artery disease, 982 (21.0)<br>Single vessel coronary artery disease, 498 (10.6)<br>Cardiac failure, 462 (9.9)<br>eGFR 60 to <90 mL/min/1.73m2, 2423 (51.7)<br>eGFR <60 mL/min/1.73m2, 1212 (25.9)<br>Urine albumin-to-creatinine ratio 30 to 300 mg/g, 1338 (28.5)<br>Urine albumin-to-creatinine ratio >300 mg/g, 509 (10.9)
		Placebo	Coronary artery disease, 1763 (75.6)<br>Multi-vessel coronary artery disease, 1100 (47.1)<br>History of myocardial infarction, 1083 (46.4)<br>History of stroke, 553 (23.7)<br>Peripheral artery disease, 479 (20.5)<br>Single vessel coronary artery disease, 238 (10.2)<br>Cardiac failure, 244 (10.5)<br>eGFR 60 to <90 mL/min/1.73m2, 1238 (53.1)<br>eGFR <60 mL/min/1.73m2, 607 (26.0)<br>Urine albumin-to-creatinine ratio 30 to 300 mg/g, 675 (28.9)<br>Urine albumin-to-creatinine ratio >300 mg/g, 260 (11.1)

Ji et al. 2014 [46]	Dapagliflozin 5 mg/day	History of dyslipidemia, 49 (38.3)<br>History of hypertension, 49 (38.3)
	Dapagliflozin 10 mg/day	History of dyslipidemia, 57 (42.9)<br>History of hypertension, 50 (37.6)
	Placebo	History of dyslipidemia, 53 (40.2)<br>History of hypertension, 54 (40.9)
Inagaki et al. 2013 [43]	Canagliflozin 50 mg/day	Retinopathy, 4 (4.9)<br>Neuropathy, 4 (4.9)<br>Nephropathy, 9 (11.0)<br>Hypertension, 34 (41.5)<br>Dyslipidaemia, 52 (63.4)
	Canagliflozin 100 mg/day	Retinopathy, 3 (4.1)<br>Neuropathy, 1 (1.4)<br>Nephropathy, 5 (6.8)<br>Hypertension, 39 (52.7)<br>Dyslipidaemia, 44 (59.5)
	Canagliflozin 200 mg/day	Retinopathy, 1 (1.3)<br>Neuropathy, 1 (1.3)<br>Nephropathy, 4 (5.3)<br>Hypertension, 33 (43.4)<br>Dyslipidaemia, 49 (64.5)
	Canagliflozin 300 mg/day	Retinopathy, 4 (5.3)<br>Neuropathy, 0 (0.0)<br>Nephropathy, 4 (5.3)<br>Hypertension, 25 (33.3)<br>Dyslipidaemia, 52 (69.3)
	Placebo	Retinopathy, 3 (4.0)<br>Neuropathy, 0 (0.0)<br>Nephropathy, 7 (9.3)<br>Hypertension, 37 (49.3)<br>Dyslipidaemia, 41 (54.7)

Table S2. The reported comorbidities of the population of the included studies; T2DM: Type 2 diabetes mellitus; T1DM: Type 1 diabetes mellitus; SGLTi / SGLTi2: Sodium-glucose cotransporter-2 inhibitor; COPD: Chronic obstructive pulmonary disease; HF: Heart failure; eGFR: Estimated glomerular filtration rate (mL/min/1.73 m^2); GFR: Glomerular filtration rate (mL/min/1.73 m^2); TIA: Transient ischemic attack. Comorbidities are reported as number of patients (n) and percentage (%).

Study ID	D1	D2	D3	D4	D5	Overall
Agarwal et al. 2025 (CONFIDENCE Trial)	+	+	+	+	+	+
Heshmat et al. 2025	!	-	+	+	+	-
Huang et al. 2025 (DEFORM Trial)	+	+	+	+	+	+
Mocan et al. 2025	!	+	+	+	!	!
Raposeiras-Roubin et al. 2025 (DapaTAVI Trial)	+	+	+	+	+	+
Snel et al. 2025	+	+	+	+	+	+
Zhou et al. 2025	+	+	+	+	!	!
Butler et al. 2024 (EMPACT-MI Trial)	+	+	+	+	+	+
James et al. 2024 (DAPA-MI Trial)	+	+	+	+	+	+
Kosiborod et al. 2024 (ACTIV-4a Trial)	+	+	+	+	+	+
Kumar et al. 2024	+	+	+	+	!	!
Li et al. 2024	+	+	+	+	+	+
Liang et al. 2024	+	+	+	+	!	!
Lin et al. 2024	+	+	+	+	+	+
McMurray et al. 2024 (DETERMINE Trial)	+	+	+	+	+	+
Pastore et al. 2024 (DAPA-ECHO Trial)	+	+	+	+	+	+
Tavares et al. 2024 (DEFENDER Trial)	+	+	+	+	+	+
Emara et al. 2023 (DAPA-RESPONSE-AHF Trial)	+	+	+	+	+	+
Liu et al. 2023	+	+	+	+	!	!
RECOVERY Collaborative Group 2023 (RECOVERY Trial)	+	+	+	+	+	+
EMPA-KIDNEY Collaborative Group 2023 (EMPA-KIDNEY)	+	+	+	+	+	+
Adel et al. 2022	+	+	+	+	!	!
Charaya et al. 2022	!	+	+	+	!	!
Reis et al. 2022	+	+	+	+	!	!
Solomon et al. 2022 (DELIVER Trial)	+	+	+	+	+	+
Von Lewinski et al. 2022 (EMMY Trial)	+	+	+	+	+	+
Voors et al. 2022 (EMPULSE Trial)	+	+	+	+	+	+
Anker et al. 2021 (EMPEROR-Preserved Trial)	+	+	+	+	+	+
Bhatt et al. 2021 (SCORED Trial)	+	+	+	+	+	+
Bhatt et al. 2021 (SOLOIST-WHF Trial)	+	+	+	+	+	+
Kosiborod et al. 2021 (DARE-19 Trial)	+	+	+	+	+	+
Lee et al. 2021 (SUGAR-DM-HF Trial)	+	+	+	+	+	+
Cannon et al. 2020 (VERTIS-CV Trial)	+	+	+	+	+	+
Damman et al. 2020 (EMPA-RESPONSE-AHF Trial)	!	+	+	+	+	!
Heerspink et al. 2020 (DAPA-CKD Trial)	+	+	+	+	+	+
Jensen et al. 2020 (EMPIRE-HF Trial)	+	+	+	+	+	+
Katakami et al. 2020 (UTOPIA Trial)	+	+	+	+	+	+
Packer et al. 2020 (EMPEROR-Reduced Trial)	+	+	+	+	+	+
Shimizu et al. 2020 (EMBODY Trial)	+	+	+	+	+	+
Kaku et al. 2019	+	+	+	+	-	-
McMurray et al. 2019 (DAPA-HF Trial)	+	+	+	+	+	+
Nassif et al. 2019 (DEFINE-HF Trial)	+	+	+	+	+	+
Perkovic et al. 2019 (CREDENCE Trial)	+	+	+	+	+	+
Wiviott et al. 2019 (DECLARE–TIMI 58 Trial)	+	+	+	+	+	+
Ikeda et al. 2015	+	+	+	+	+	+
Kovacs et al. 2015	+	+	+	+	+	+
Zinman et al. 2015 (EMPA-REG OUTCOME Trial)	+	+	+	+	+	+
Ji et al. 2014	+	+	+	+	+	+
Inagaki et al. 2013	!	+	+	+	+	!
Berg et al. 2025 (DAPA ACT HF-TIMI 68 Trial)	+	+	+	+	+	+

+ Low risk
! Some concerns
- High risk

D1 Randomisation process
D2 Deviations from the intended interventic
D3 Missing outcome data
D4 Measurement of the outcome
D5 Selection of the reported result

FigureS1. Risk of bias 2 (RoB2) figure.

Study or Subgroup	SGLT2i Events	SGLT2i Total	Control Events	Control Total	Weight	Risk Ratio M-H, Random, 95% CI	Year
Inagaki et al. 2013	0	307	0	75		Not estimable	2013
Ji et al. 2014	0	261	0	132		Not estimable	2014
Kovacs et al. 2015	4	333	1	165	0.2%	1.98 [0.22, 17.59]	2015
Ikeda et al. 2015	0	328	0	66		Not estimable	2015
Kaku et al. 2019	0	115	0	59		Not estimable	2019
Nassif et al. 2019 (DEFINE-HF Trial)	1	131	1	132	0.1%	1.01 [0.06, 15.94]	2019
Shimizu et al. 2020 (EMBODY Trial)	0	46	0	50		Not estimable	2020
Damman et al. 2020 (EMPA-RESPONSE-AHF Trial)	1	40	3	39	0.2%	0.33 [0.04, 2.99]	2020
Jensen et al. 2020 (EMPIRE-HF Trial)	0	95	0	95		Not estimable	2020
Kosiborod et al. 2021 (DARE-19 Trial)	41	625	54	625	7.0%	0.76 [0.51, 1.12]	2021
Lee et al. 2021 (SUGAR-DM-HF Trial)	2	52	0	53	0.1%	5.09 [0.25, 103.62]	2021
Bhatt et al. 2021 (SOLOIST-WHF Trial)	65	608	76	614	11.0%	0.86 [0.63, 1.18]	2021
Reis et al. 2022	0	20	0	20		Not estimable	2022
Von Lewinski et al. 2022 (EMMY Trial)	3	237	0	239	0.1%	7.06 [0.37, 135.92]	2022
Voors et al. 2022 (EMPULSE Trial)	11	265	22	265	2.1%	0.50 [0.25, 1.01]	2022
Charaya et al. 2022	9	44	12	44	1.9%	0.75 [0.35, 1.60]	2022
Liu et al. 2023	1	52	7	53	0.3%	0.15 [0.02, 1.14]	2023
RECOVERY Collaborative Group 2023 (RECOVERY Trial)	289	2113	307	2158	47.9%	0.96 [0.83, 1.12]	2023
Emara et al. 2023 (DAPA-RESPONSE-AHF Trial)	6	45	9	42	1.2%	0.62 [0.24, 1.60]	2023
McMurray et al. 2024 (DETERMINE-reduced Trial)	3	156	2	157	0.3%	1.51 [0.26, 8.91]	2024
James et al. 2024 (DAPA-MI Trial)	41	2019	33	1998	5.2%	1.23 [0.78, 1.94]	2024
Kosiborod et al. 2024 (ACTIV-4a Trial)	37	285	42	288	6.3%	0.89 [0.59, 1.34]	2024
Kumar et al. 2024	10	200	4	50	0.9%	0.63 [0.20, 1.91]	2024
Liang et al. 2024	1	30	3	30	0.2%	0.33 [0.04, 3.03]	2024
Li et al. 2024	0	27	0	9		Not estimable	2024
McMurray et al. 2024 (DETERMINE-preserved Trial)	3	253	2	251	0.3%	1.49 [0.25, 8.83]	2024
Mocan et al. 2025	1	49	2	49	0.2%	0.50 [0.05, 5.34]	2025
Raposeiras-Roubin et al. 2025 (DapaTAVI Trial)	47	605	55	617	7.7%	0.87 [0.60, 1.27]	2025
Snel et al. 2025	1	25	0	30	0.1%	3.58 [0.15, 84.13]	2025
Zhou et al. 2025	0	50	3	48	0.1%	0.14 [0.01, 2.59]	2025
Agarwal et al. 2025 (CONFIDENCE Trial)	3	268	0	264	0.1%	6.90 [0.36, 132.85]	2025
Heshmat et al. 2025	1	27	1	27	0.1%	1.00 [0.07, 15.18]	2025
Huang et al. 2025 (DEFORM Trial)	2	52	1	52	0.2%	2.00 [0.19, 21.38]	2025
Berg et al. 2025 (DAPA ACT HF-TIMI 68 Trial)	36	1218	53	1183	6.2%	0.66 [0.44, 1.00]	2025
**Total (95% CI)**		**10981**		**9979**	**100.0%**	**0.89 [0.80, 0.99]**	
Total events	619		693				

Heterogeneity: Tau² = 0.00; Chi² = 23.07, df = 25 (P = 0.57); I² = 0%
Test for overall effect: Z = 2.22 (P = 0.03)

Risk Ratio M-H, Random, 95% CI

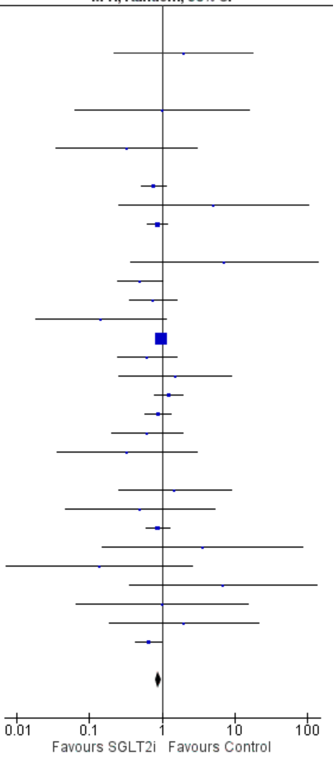

Figure S2. Forest plot of all-cause mortality up to one-year.

Study or Subgroup	SGLT2i Events	SGLT2i Total	Control Events	Control Total	Weight	Risk Ratio M-H, Random, 95% CI	Year
Inagaki et al. 2013	0	307	0	75		Not estimable	2013
Ji et al. 2014	0	261	0	132		Not estimable	2014
Kovacs et al. 2015	4	333	1	165	0.2%	1.98 [0.22, 17.59]	2015
Ikeda et al. 2015	0	328	0	66		Not estimable	2015
Kaku et al. 2019	0	115	0	59		Not estimable	2019
Nassif et al. 2019 (DEFINE-HF Trial)	1	131	1	132	0.1%	1.01 [0.06, 15.94]	2019
Shimizu et al. 2020 (EMBODY Trial)	0	46	0	50		Not estimable	2020
Damman et al. 2020 (EMPA-RESPONSE-AHF Trial)	1	40	3	39	0.2%	0.33 [0.04, 2.99]	2020
Jensen et al. 2020 (EMPIRE-HF Trial)	0	95	0	95		Not estimable	2020
Kosiborod et al. 2021 (DARE-19 Trial)	41	625	54	625	7.0%	0.76 [0.51, 1.12]	2021
Lee et al. 2021 (SUGAR-DM-HF Trial)	2	52	0	53	0.1%	5.09 [0.25, 103.62]	2021
Bhatt et al. 2021 (SOLOIST-WHF Trial)	65	608	76	614	11.0%	0.86 [0.63, 1.18]	2021
Reis et al. 2022	0	20	0	20		Not estimable	2022
Von Lewinski et al. 2022 (EMMY Trial)	3	237	0	239	0.1%	7.06 [0.37, 135.92]	2022
Voors et al. 2022 (EMPULSE Trial)	11	265	22	265	2.2%	0.50 [0.25, 1.01]	2022
Charaya et al. 2022	9	44	12	44	1.9%	0.75 [0.35, 1.60]	2022
Liu et al. 2023	1	52	7	53	0.3%	0.15 [0.02, 1.14]	2023
RECOVERY Collaborative Group 2023 (RECOVERY Trial)	289	2113	307	2158	48.0%	0.96 [0.83, 1.12]	2023
Emara et al. 2023 (DAPA-RESPONSE-AHF Trial)	6	45	9	42	1.2%	0.62 [0.24, 1.60]	2023
McMurray et al. 2024 (DETERMINE-reduced Trial)	3	156	2	157	0.3%	1.51 [0.26, 8.91]	2024
James et al. 2024 (DAPA-MI Trial)	41	2019	33	1998	5.2%	1.23 [0.78, 1.94]	2024
Kosiborod et al. 2024 (ACTIV-4a Trial)	37	285	42	288	6.3%	0.89 [0.59, 1.34]	2024
Kumar et al. 2024	10	200	4	50	0.9%	0.63 [0.20, 1.91]	2024
Liang et al. 2024	1	30	3	30	0.2%	0.33 [0.04, 3.03]	2024
Li et al. 2024	0	27	0	9		Not estimable	2024
McMurray et al. 2024 (DETERMINE-preserved Trial)	3	253	2	251	0.3%	1.49 [0.25, 8.83]	2024
Mocan et al. 2025	1	49	2	49	0.2%	0.50 [0.05, 5.34]	2025
Raposeiras-Roubin et al. 2025 (DapaTAVI Trial)	47	605	55	617	7.7%	0.87 [0.60, 1.27]	2025
Snel et al. 2025	1	25	0	30	0.1%	3.58 [0.15, 84.13]	2025
Zhou et al. 2025	0	50	3	48	0.1%	0.14 [0.01, 2.59]	2025
Agarwal et al. 2025 (CONFIDENCE Trial)	3	268	0	264	0.1%	6.90 [0.36, 132.85]	2025
Heshmat et al. 2025	1	27	1	27	0.0%	1.00 [0.07, 15.18]	2025
Huang et al. 2025 (DEFORM Trial)	2	52	1	52	0.2%	2.00 [0.19, 21.38]	2025
Berg et al. 2025 (DAPA ACT HF-TIMI 68 Trial)	36	1218	53	1183	6.2%	0.66 [0.44, 1.00]	2025
**Total (95% CI)**		**10954**		**9952**	**100.0%**	**0.89 [0.80, 0.99]**	
Total events	618		692				

Heterogeneity: Tau² = 0.00; Chi² = 23.06, df = 24 (P = 0.52); I² = 0%
Test for overall effect: Z = 2.22 (P = 0.03)

Risk Ratio M-H, Random, 95% CI

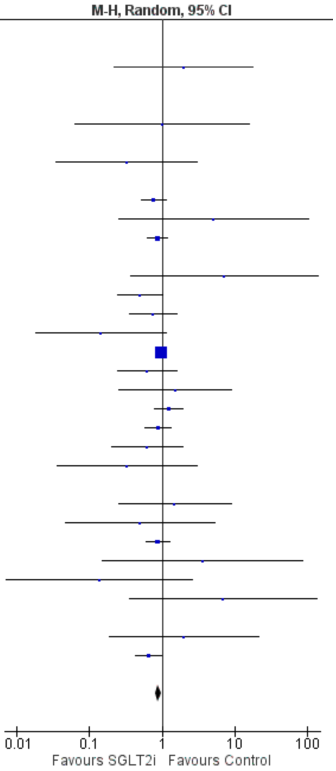

Figure S3. Sensitivity analysis of all-cause mortality up to one-year, excluding high risk of bias studies.

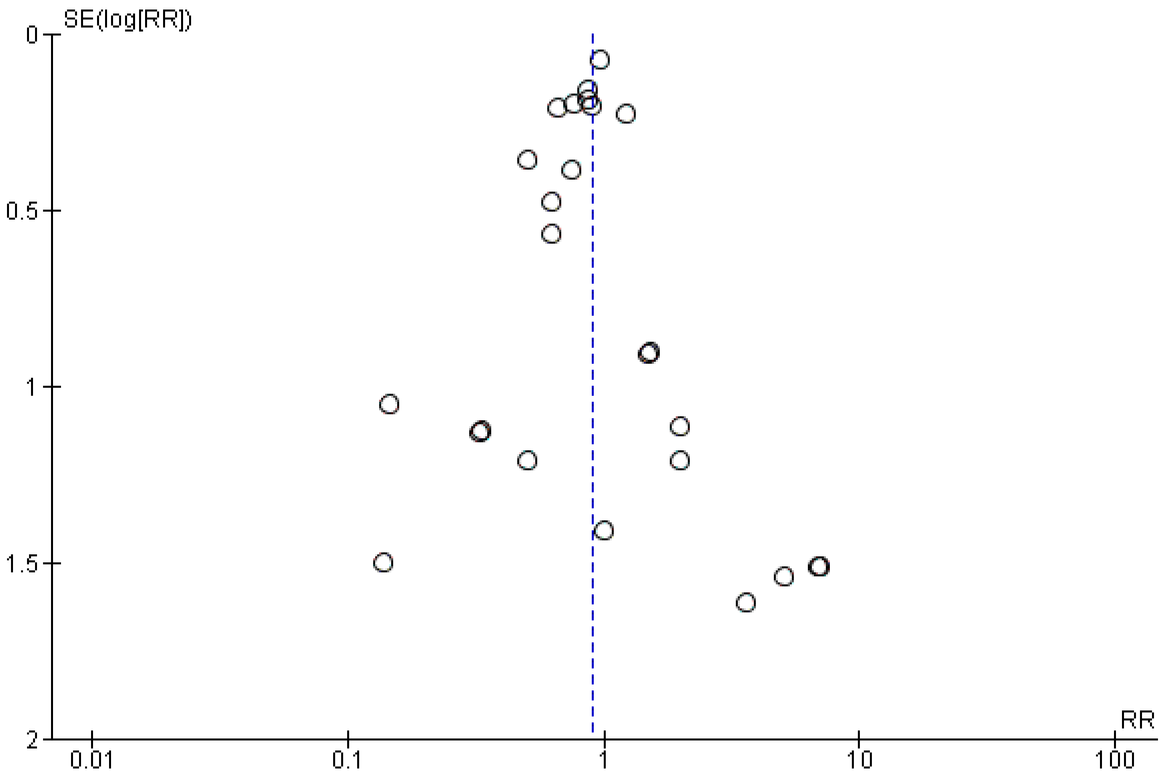

Figure S4. Funnel plot of all-cause mortality up to one year.

Study or Subgroup	SGLT2i Events	SGLT2i Total	Control Events	Control Total	Weight	Risk Ratio M-H, Random, 95% CI	Year
**1.3.1 Acute cardiac decompensation**							
Shimizu et al. 2020 (EMBODY Trial)	0	46	0	50		Not estimable	2020
Damman et al. 2020 (EMPA-RESPONSE-AHF Trial)	1	40	3	39	1.1%	0.33 [0.04, 2.99]	2020
Bhatt et al. 2021 (SOLOIST-WHF Trial)	65	608	76	614	29.4%	0.86 [0.63, 1.18]	2021
Von Lewinski et al. 2022 (EMMY Trial)	3	237	0	239	0.6%	7.06 [0.37, 135.92]	2022
Voors et al. 2022 (EMPULSE Trial)	11	265	22	265	9.7%	0.50 [0.25, 1.01]	2022
Charaya et al. 2022	9	44	12	44	8.5%	0.75 [0.35, 1.60]	2022
Liu et al. 2023	1	52	7	53	1.3%	0.15 [0.02, 1.14]	2023
Emara et al. 2023 (DAPA-RESPONSE-AHF Trial)	6	45	9	42	5.8%	0.62 [0.24, 1.60]	2023
Liang et al. 2024	1	30	3	30	1.2%	0.33 [0.04, 3.03]	2024
James et al. 2024 (DAPA-MI Trial)	41	2019	33	1998	18.8%	1.23 [0.78, 1.94]	2024
Mocan et al. 2025	1	49	2	49	1.0%	0.50 [0.05, 5.34]	2025
Berg et al. 2025 (DAPA ACT HF-TIMI 68 Trial)	36	1218	53	1183	21.2%	0.66 [0.44, 1.00]	2025
Zhou et al. 2025	0	50	3	48	0.7%	0.14 [0.01, 2.59]	2025
Heshmat et al. 2025	1	27	1	27	0.8%	1.00 [0.07, 15.18]	2025
**Subtotal (95% CI)**		**4730**		**4681**	**100.0%**	**0.76 [0.60, 0.97]**	
Total events	176		224				
Heterogeneity: Tau² = 0.03; Chi² = 14.02, df = 12 (P = 0.30); I² = 14%							
Test for overall effect: Z = 2.21 (P = 0.03)							
**1.3.2 Chronic HF**							
Nassif et al. 2019 (DEFINE-HF Trial)	1	131	1	132	15.0%	1.01 [0.06, 15.94]	2019
Jensen et al. 2020 (EMPIRE-HF Trial)	0	95	0	95		Not estimable	2020
Lee et al. 2021 (SUGAR-DM-HF Trial)	2	52	0	53	12.6%	5.09 [0.25, 103.62]	2021
Reis et al. 2022	0	20	0	20		Not estimable	2022
McMurray et al. 2024 (DETERMINE-preserved Trial)	3	253	2	251	36.1%	1.49 [0.25, 8.83]	2024
McMurray et al. 2024 (DETERMINE-reduced Trial)	3	156	2	157	36.3%	1.51 [0.26, 8.91]	2024
**Subtotal (95% CI)**		**707**		**708**	**100.0%**	**1.65 [0.57, 4.80]**	
Total events	9		5				
Heterogeneity: Tau² = 0.00; Chi² = 0.69, df = 3 (P = 0.88); I² = 0%							
Test for overall effect: Z = 0.91 (P = 0.36)							
**1.3.3 DM / high-risk DM**							
Inagaki et al. 2013	0	307	0	75		Not estimable	2013
Ji et al. 2014	0	261	0	132		Not estimable	2014
Kovacs et al. 2015	4	333	1	165	100.0%	1.98 [0.22, 17.59]	2015
Ikeda et al. 2015	0	328	0	66		Not estimable	2015
Kaku et al. 2019	0	115	0	59		Not estimable	2019
Li et al. 2024	0	27	0	9		Not estimable	2024
**Subtotal (95% CI)**		**1371**		**506**	**100.0%**	**1.98 [0.22, 17.59]**	
Total events	4		1				
Heterogeneity: Not applicable							
Test for overall effect: Z = 0.61 (P = 0.54)							
**1.3.4 DM with established CVD**							
Kumar et al. 2024	10	200	4	50	100.0%	0.63 [0.20, 1.91]	2024
**Subtotal (95% CI)**		**200**		**50**	**100.0%**	**0.63 [0.20, 1.91]**	
Total events	10		4				
Heterogeneity: Not applicable							
Test for overall effect: Z = 0.82 (P = 0.41)							
**1.3.5 COVID-19 / critical illness**							
Kosiborod et al. 2021 (DARE-19 Trial)	41	625	54	625	11.4%	0.76 [0.51, 1.12]	2021
RECOVERY Collaborative Group 2023 (RECOVERY Trial)	289	2113	307	2158	78.3%	0.96 [0.83, 1.12]	2023
Kosiborod et al. 2024 (ACTIV-4a Trial)	37	285	42	288	10.3%	0.89 [0.59, 1.34]	2024
**Subtotal (95% CI)**		**3023**		**3071**	**100.0%**	**0.93 [0.81, 1.06]**	
Total events	367		403				
Heterogeneity: Tau² = 0.00; Chi² = 1.27, df = 2 (P = 0.53); I² = 0%							
Test for overall effect: Z = 1.10 (P = 0.27)							
**1.3.6 Cardiac procedure**							
Raposeiras-Roubin et al. 2025 (DapaTAVI Trial)	47	605	55	617	98.6%	0.87 [0.60, 1.27]	2025
Snel et al. 2025	1	25	0	30	1.4%	3.58 [0.15, 84.13]	2025
**Subtotal (95% CI)**		**630**		**647**	**100.0%**	**0.89 [0.61, 1.29]**	
Total events	48		55				
Heterogeneity: Tau² = 0.00; Chi² = 0.76, df = 1 (P = 0.38); I² = 0%							
Test for overall effect: Z = 0.63 (P = 0.53)							
**1.3.7 Functional MR**							
Huang et al. 2025 (DEFORM Trial)	2	52	1	52	100.0%	2.00 [0.19, 21.38]	2025
**Subtotal (95% CI)**		**52**		**52**	**100.0%**	**2.00 [0.19, 21.38]**	
Total events	2		1				
Heterogeneity: Not applicable							
Test for overall effect: Z = 0.57 (P = 0.57)							
**1.3.8 CKD**							
Agarwal et al. 2025 (CONFIDENCE Trial)	3	268	0	264	100.0%	6.90 [0.36, 132.85]	2025
**Subtotal (95% CI)**		**268**		**264**	**100.0%**	**6.90 [0.36, 132.85]**	
Total events	3		0				
Heterogeneity: Not applicable							
Test for overall effect: Z = 1.28 (P = 0.20)							

Test for subgroup differences: Chi² = 6.41, df = 7 (P = 0.49), I² = 0%

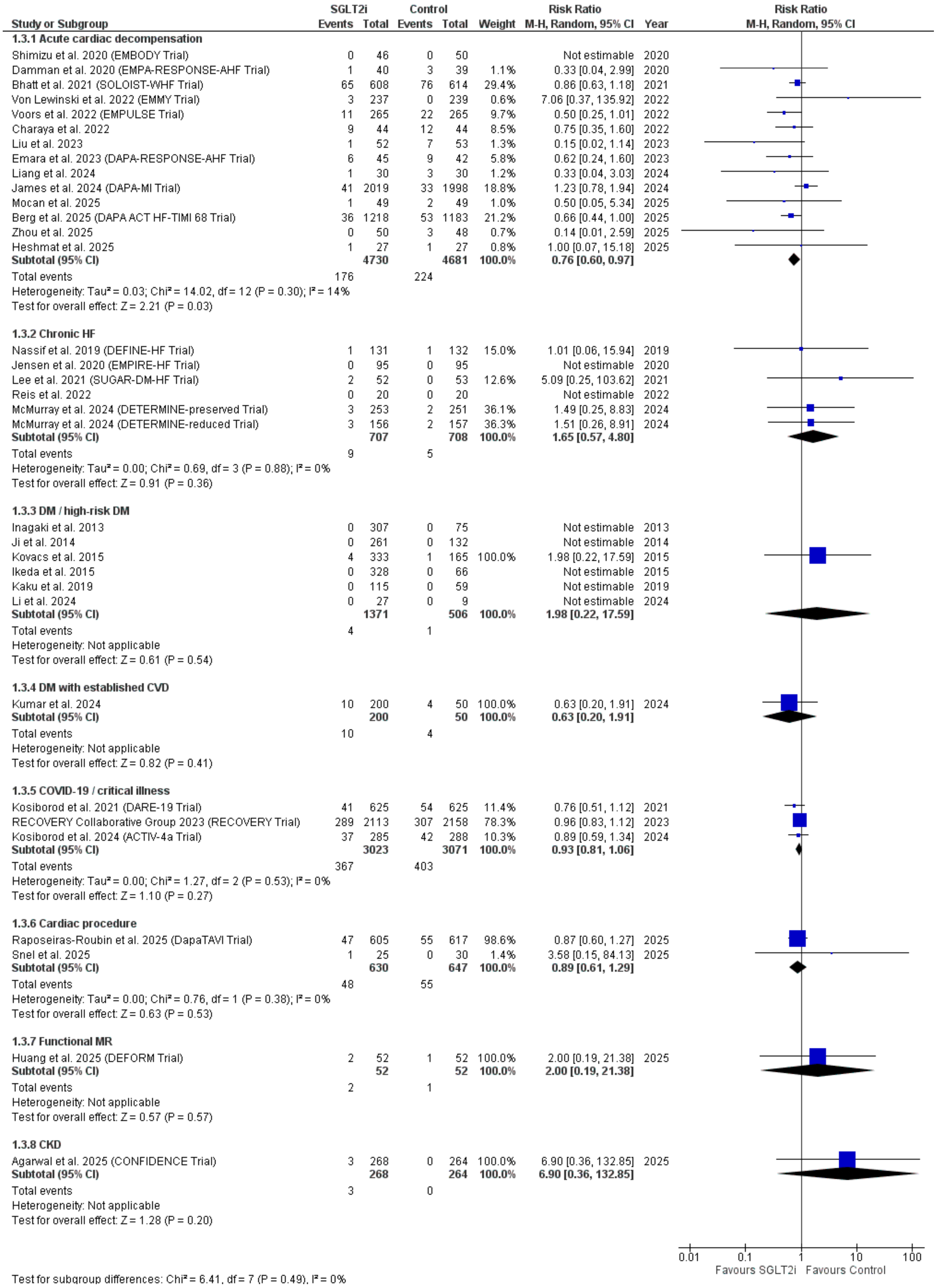

Figure S5. Subgroup analysis based on population for all-cause mortality up to one-year.

Study or Subgroup	SGLT2i Events	SGLT2i Total	Control Events	Control Total	Weight	Risk Ratio M-H, Random, 95% CI	Year
**1.2.1 Dapagliflozin**							
Ji et al. 2014	0	261	0	132		Not estimable	2014
Nassif et al. 2019 (DEFINE-HF Trial)	1	131	1	132	0.5%	1.01 [0.06, 15.94]	2019
Kosiborod et al. 2021 (DARE-19 Trial)	41	625	54	625	22.7%	0.76 [0.51, 1.12]	2021
Charaya et al. 2022	9	44	12	44	6.1%	0.75 [0.35, 1.60]	2022
Reis et al. 2022	0	20	0	20		Not estimable	2022
Emara et al. 2023 (DAPA-RESPONSE-AHF Trial)	6	45	9	42	3.9%	0.62 [0.24, 1.60]	2023
James et al. 2024 (DAPA-MI Trial)	41	2019	33	1998	16.8%	1.23 [0.78, 1.94]	2024
Liang et al. 2024	1	30	3	30	0.7%	0.33 [0.04, 3.03]	2024
Li et al. 2024 (Dapagliflozin)	0	9	0	9		Not estimable	2024
McMurray et al. 2024 (DETERMINE-preserved Trial)	3	253	2	251	1.1%	1.49 [0.25, 8.83]	2024
McMurray et al. 2024 (DETERMINE-reduced Trial)	3	156	2	157	1.1%	1.51 [0.26, 8.91]	2024
Berg et al. 2025 (DAPA ACT HF-TIMI 68 Trial)	36	1218	53	1183	20.1%	0.66 [0.44, 1.00]	2025
Zhou et al. 2025	0	50	3	48	0.4%	0.14 [0.01, 2.59]	2025
Heshmat et al. 2025	1	27	1	27	0.5%	1.00 [0.07, 15.18]	2025
Huang et al. 2025 (DEFORM Trial)	2	52	1	52	0.6%	2.00 [0.19, 21.38]	2025
Mocan et al. 2025	1	49	2	49	0.6%	0.50 [0.05, 5.34]	2025
Raposeiras-Roubin et al. 2025 (DapaTAVI Trial)	47	605	55	617	25.0%	0.87 [0.60, 1.27]	2025
**Subtotal (95% CI)**		**5594**		**5416**	**100.0%**	**0.83 [0.69, 1.00]**	
Total events	192		231				
Heterogeneity: Tau² = 0.00; Chi² = 8.43, df = 13 (P = 0.81); I² = 0%							
Test for overall effect: Z = 1.98 (P = 0.05)							
**1.2.2 Empagliflozin**							
Kovacs et al. 2015	4	333	1	165	4.7%	1.98 [0.22, 17.59]	2015
Damman et al. 2020 (EMPA-RESPONSE-AHF Trial)	1	40	3	39	4.5%	0.33 [0.04, 2.99]	2020
Jensen et al. 2020 (EMPIRE-HF Trial)	0	95	0	95		Not estimable	2020
Shimizu et al. 2020 (EMBODY Trial)	0	46	0	50		Not estimable	2020
Lee et al. 2021 (SUGAR-DM-HF Trial)	2	52	0	53	2.6%	5.09 [0.25, 103.62]	2021
Von Lewinski et al. 2022 (EMMY Trial)	3	237	0	239	2.7%	7.06 [0.37, 135.92]	2022
Voors et al. 2022 (EMPULSE Trial)	11	265	22	265	22.6%	0.50 [0.25, 1.01]	2022
RECOVERY Collaborative Group 2023 (RECOVERY Trial)	289	2113	307	2158	39.2%	0.96 [0.83, 1.12]	2023
Liu et al. 2023	1	52	7	53	5.2%	0.15 [0.02, 1.14]	2023
Kumar et al. 2024	10	200	4	50	13.5%	0.63 [0.20, 1.91]	2024
Agarwal et al. 2025 (CONFIDENCE Trial)	3	268	0	264	2.7%	6.90 [0.36, 132.85]	2025
Snel et al. 2025	1	25	0	30	2.4%	3.58 [0.15, 84.13]	2025
**Subtotal (95% CI)**		**3726**		**3461**	**100.0%**	**0.84 [0.51, 1.38]**	
Total events	325		344				
Heterogeneity: Tau² = 0.16; Chi² = 13.59, df = 9 (P = 0.14); I² = 34%							
Test for overall effect: Z = 0.69 (P = 0.49)							
**1.2.3 Canagliflozin**							
Inagaki et al. 2013	0	307	0	75		Not estimable	2013
**Subtotal (95% CI)**		**307**		**75**		**Not estimable**	
Total events	0		0				
Heterogeneity: Not applicable							
Test for overall effect: Not applicable							
**1.2.4 Ipragliflozin**							
Kaku et al. 2019	0	115	0	59		Not estimable	2019
**Subtotal (95% CI)**		**115**		**59**		**Not estimable**	
Total events	0		0				
Heterogeneity: Not applicable							
Test for overall effect: Not applicable							
**1.2.5 Janagliflozin**							
Li et al. 2024 (Janagliflozin)	0	18	0	9		Not estimable	2024
**Subtotal (95% CI)**		**18**		**9**		**Not estimable**	
Total events	0		0				
Heterogeneity: Not applicable							
Test for overall effect: Not applicable							
**1.2.6 Sotagliflozin**							
Bhatt et al. 2021 (SOLOIST-WHF Trial)	65	608	76	614	100.0%	0.86 [0.63, 1.18]	2021
**Subtotal (95% CI)**		**608**		**614**	**100.0%**	**0.86 [0.63, 1.18]**	
Total events	65		76				
Heterogeneity: Not applicable							
Test for overall effect: Z = 0.92 (P = 0.36)							
**1.2.7 Tofogliflozin**							
Ikeda et al. 2015	0	328	0	66		Not estimable	2015
**Subtotal (95% CI)**		**328**		**66**		**Not estimable**	
Total events	0		0				
Heterogeneity: Not applicable							
Test for overall effect: Not applicable							
**1.2.8 Multiple SGLT2i (dapagliflozin, empagliflozin, canagliflozin or ertugliflozin)**							
Kosiborod et al. 2024 (ACTIV-4a Trial)	37	285	42	288	100.0%	0.89 [0.59, 1.34]	2024
**Subtotal (95% CI)**		**285**		**288**	**100.0%**	**0.89 [0.59, 1.34]**	
Total events	37		42				
Heterogeneity: Not applicable							
Test for overall effect: Z = 0.56 (P = 0.58)							

Test for subgroup differences: Chi² = 0.13, df = 3 (P = 0.99), I² = 0%

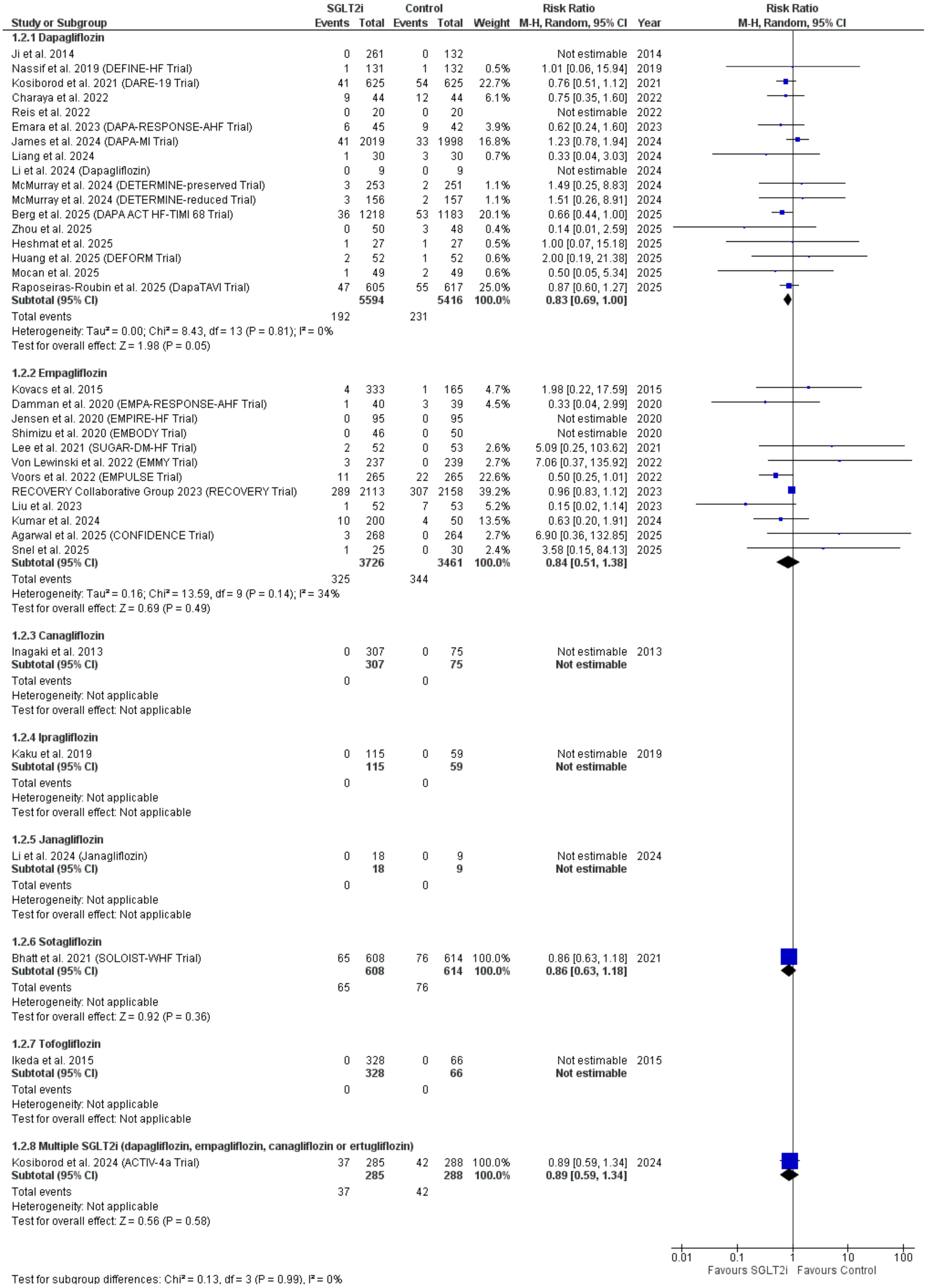

Figure S6. Subgroup analysis based on the adopted SGLT2 inhibitors for all-cause mortality up to one-year.

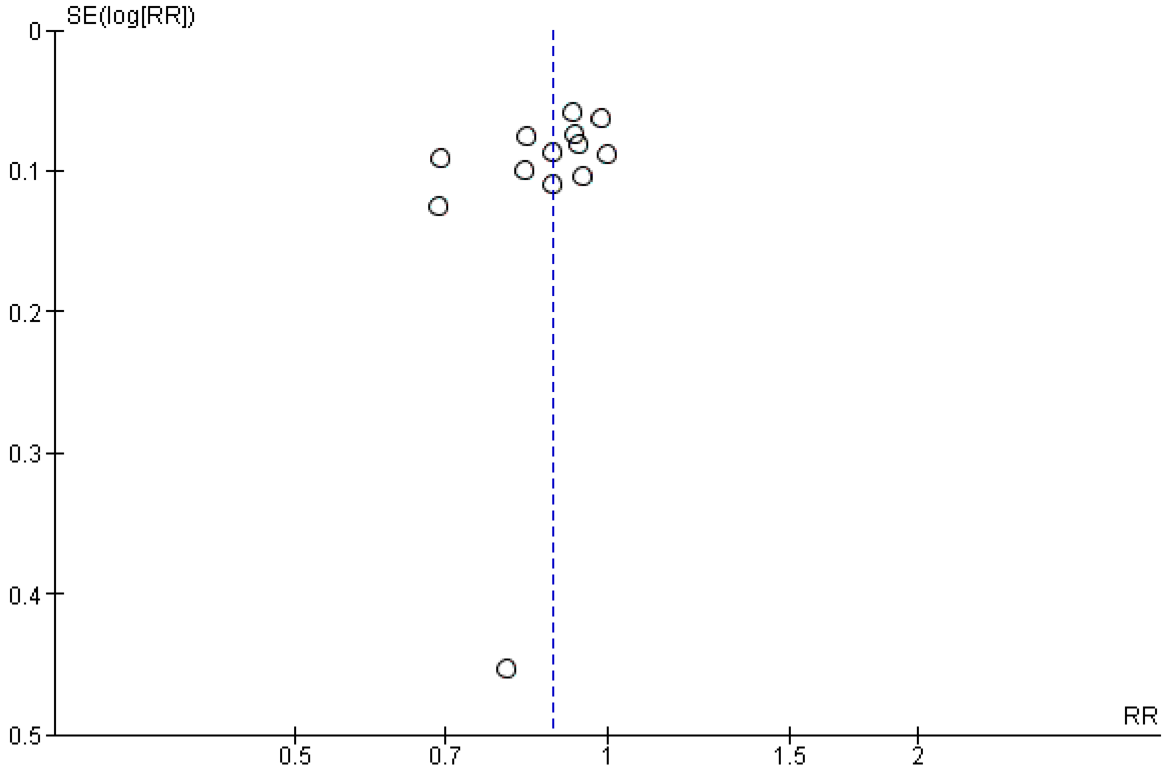

Figure S7. Funnel plot of all-cause mortality more than one year.

Study or Subgroup	SGLTi Events	SGLTi Total	Control Events	Control Total	Weight	Risk Ratio M-H, Random, 95% CI	Year
Zinman et al. 2015 (EMPA-REG OUTCOME Trial)	172	4687	137	2333	5.8%	0.62 [0.50, 0.78]	2015
McMurray et al. 2019 (DAPA-HF Trial)	227	2373	273	2371	10.0%	0.83 [0.70, 0.98]	2019
Nassif et al. 2019 (DEFINE-HF Trial)	1	131	1	132	0.0%	1.01 [0.06, 15.94]	2019
Perkovic et al. 2019 (CREDENCE Trial)	110	2202	140	2199	4.7%	0.78 [0.62, 1.00]	2019
Wiviott et al. 2019 (DECLARE-TIMI 58 Trial)	245	8582	249	8578	9.2%	0.98 [0.83, 1.17]	2019
Cannon et al. 2020 (VERTIS-CV Trial)	341	5499	184	2747	9.3%	0.93 [0.78, 1.10]	2020
Damman et al. 2020 (EMPA-RESPONSE-AHF Trial)	1	40	3	39	0.1%	0.33 [0.04, 2.99]	2020
Heerspink et al. 2020 (DAPA-CKD Trial)	65	2152	80	2152	2.7%	0.81 [0.59, 1.12]	2020
Katakami et al. 2020 (UTOPIA Trial)	0	169	0	171		Not estimable	2020
Packer et al. 2020 (EMPEROR-Reduced Trial)	187	1863	202	1867	7.9%	0.93 [0.77, 1.12]	2020
Anker et al. 2021 (EMPEROR-Preserved Trial)	219	2997	244	2991	9.1%	0.90 [0.75, 1.07]	2021
Bhatt et al. 2021 (SCORED Trial)	155	5292	170	5292	6.1%	0.91 [0.74, 1.13]	2021
Bhatt et al. 2021 (SOLOIST-WHF Trial)	51	608	58	614	2.2%	0.89 [0.62, 1.27]	2021
Lee et al. 2021 (SUGAR-DM-HF Trial)	1	52	0	53	0.0%	3.06 [0.13, 73.36]	2021
Adel et al. 2022	1	45	2	48	0.0%	0.53 [0.05, 5.68]	2022
Solomon et al. 2022 (DELIVER Trial)	497	3131	526	3132	22.1%	0.95 [0.84, 1.06]	2022
Von Lewinski et al. 2022 (EMMY Trial)	2	237	0	239	0.0%	5.04 [0.24, 104.47]	2022
EMPA-KIDNEY Collaborative Group 2023 (EMPA-KIDNEY)	59	3304	69	3305	2.4%	0.86 [0.61, 1.21]	2023
Butler et al. 2024 (EMPACT-MI Trial)	132	3260	131	3262	5.0%	1.01 [0.80, 1.28]	2024
James et al. 2024 (DAPA-MI Trial)	27	2019	23	1998	0.9%	1.16 [0.67, 2.02]	2024
Kumar et al. 2024	6	200	3	50	0.2%	0.50 [0.13, 1.93]	2024
Pastore et al. 2024 (DAPA-ECHO Trial)	0	44	0	44		Not estimable	2024
Huang et al. 2025 (DEFORM Trial)	2	52	1	52	0.0%	2.00 [0.19, 21.38]	2025
Raposeiras-Roubin et al. 2025 (DapaTAVI Trial)	27	605	33	617	1.1%	0.83 [0.51, 1.37]	2025
Berg et al. 2025 (DAPA ACT HF-TIMI 68 Trial)	30	1218	37	1183	1.2%	0.79 [0.49, 1.27]	2025
**Total (95% CI)**		**50762**		**45469**	**100.0%**	**0.89 [0.84, 0.94]**	
Total events	2558		2566				

Heterogeneity: $Tau^2 = 0.00$; $Chi^2 = 21.13$, df = 22 (P = 0.51); $I^2 = 0\%$
Test for overall effect: Z = 4.31 (P < 0.0001)

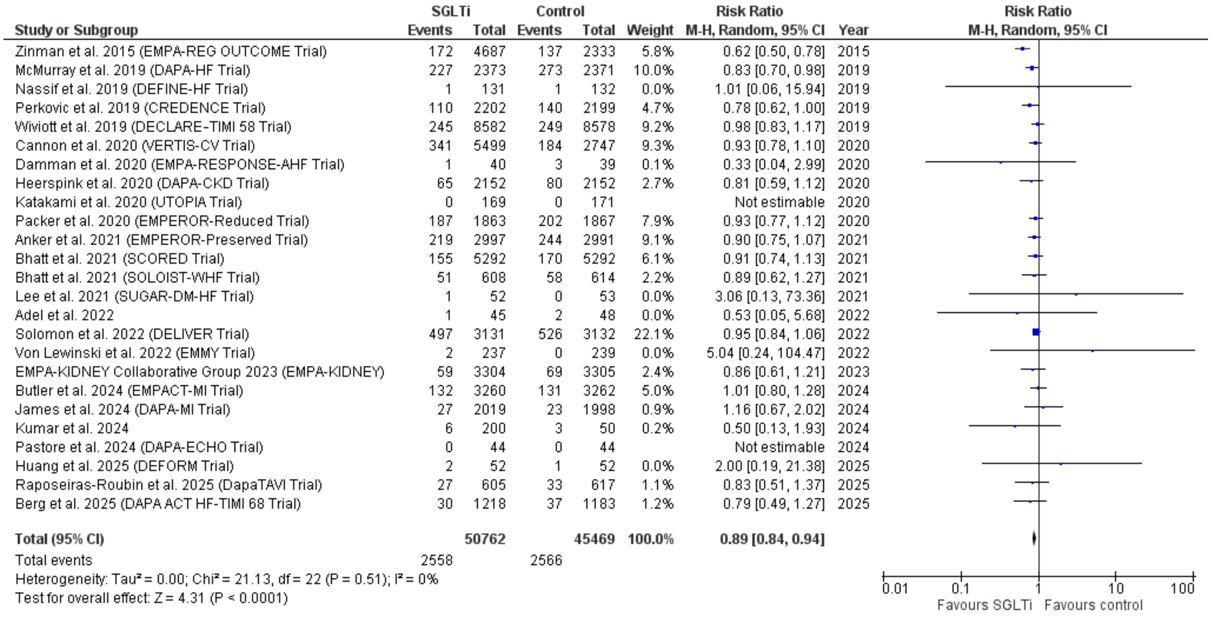

Figure S8. Forest plot of cardiovascular mortality.

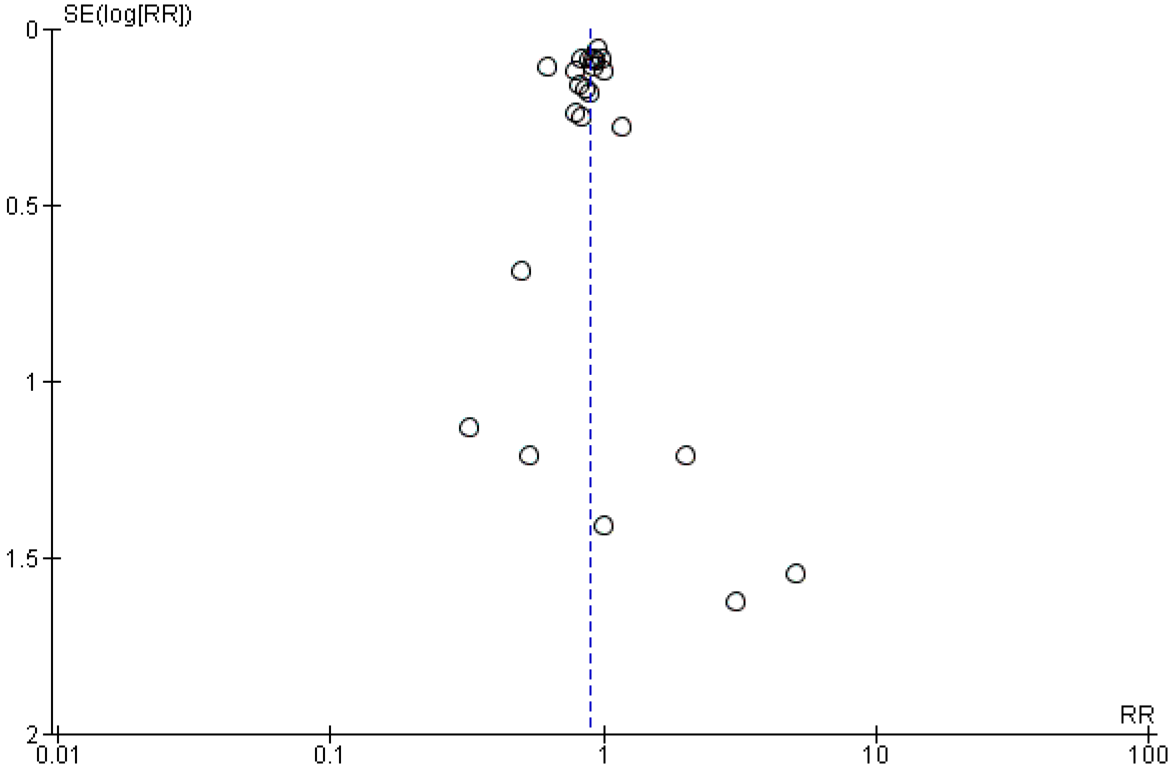

Figure S9. Funnel plot of cardiovascular mortality.

Study or Subgroup	SGLT2i Events	SGLT2i Total	Control Events	Control Total	Weight	Risk Ratio M-H, Random, 95% CI	Year
**1.12.1 Acute cardiac decompensation**							
Damman et al. 2020 (EMPA-RESPONSE-AHF Trial)	1	40	3	39	0.6%	0.33 [0.04, 2.99]	2020
Bhatt et al. 2021 (SOLOIST-WHF Trial)	51	608	58	614	22.9%	0.89 [0.62, 1.27]	2021
Adel et al. 2022	1	45	2	48	0.5%	0.53 [0.05, 5.68]	2022
Von Lewinski et al. 2022 (EMMY Trial)	2	237	0	239	0.3%	5.04 [0.24, 104.47]	2022
Butler et al. 2024 (EMPACT-MI Trial)	132	3260	131	3262	52.8%	1.01 [0.80, 1.28]	2024
James et al. 2024 (DAPA-MI Trial)	27	2019	23	1998	9.7%	1.16 [0.67, 2.02]	2024
Berg et al. 2025 (DAPA ACT HF-TIMI 68 Trial)	30	1218	37	1183	13.1%	0.79 [0.49, 1.27]	2025
**Subtotal (95% CI)**		**7427**		**7383**	**100.0%**	**0.96 [0.81, 1.14]**	
Total events	244		254				
Heterogeneity: Tau² = 0.00; Chi² = 3.77, df = 6 (P = 0.71); I² = 0%							
Test for overall effect: Z = 0.51 (P = 0.61)							
**1.12.2 Chronic HF**							
McMurray et al. 2019 (DAPA-HF Trial)	227	2373	273	2371	20.4%	0.83 [0.70, 0.98]	2019
Nassif et al. 2019 (DEFINE-HF Trial)	1	131	1	132	0.1%	1.01 [0.06, 15.94]	2019
Packer et al. 2020 (EMPEROR-Reduced Trial)	187	1863	202	1867	16.0%	0.93 [0.77, 1.12]	2020
Anker et al. 2021 (EMPEROR-Preserved Trial)	219	2997	244	2991	18.4%	0.90 [0.75, 1.07]	2021
Lee et al. 2021 (SUGAR-DM-HF Trial)	1	52	0	53	0.1%	3.06 [0.13, 73.36]	2021
Solomon et al. 2022 (DELIVER Trial)	497	3131	526	3132	45.0%	0.95 [0.84, 1.06]	2022
Pastore et al. 2024 (DAPA-ECHO Trial)	0	44	0	44		Not estimable	2024
**Subtotal (95% CI)**		**10591**		**10590**	**100.0%**	**0.91 [0.84, 0.98]**	
Total events	1132		1246				
Heterogeneity: Tau² = 0.00; Chi² = 2.22, df = 5 (P = 0.82); I² = 0%							
Test for overall effect: Z = 2.47 (P = 0.01)							
**1.12.3 DM / high-risk DM**							
Wiviott et al. 2019 (DECLARE-TIMI 58 Trial)	245	8582	249	8578	100.0%	0.98 [0.83, 1.17]	2019
Katakami et al. 2020 (UTOPIA Trial)	0	169	0	171		Not estimable	2020
**Subtotal (95% CI)**		**8751**		**8749**	**100.0%**	**0.98 [0.83, 1.17]**	
Total events	245		249				
Heterogeneity: Not applicable							
Test for overall effect: Z = 0.19 (P = 0.85)							
**1.12.4 DM with established CVD**							
Zinman et al. 2015 (EMPA-REG OUTCOME Trial)	172	4687	137	2333	97.4%	0.62 [0.50, 0.78]	2015
Cannon et al. 2020 (VERTIS-CV Trial)	341	5499	184	2747	0.0%	0.93 [0.78, 1.10]	2020
Kumar et al. 2024	6	200	3	50	2.6%	0.50 [0.13, 1.93]	2024
**Subtotal (95% CI)**		**4887**		**2383**	**100.0%**	**0.62 [0.50, 0.77]**	
Total events	178		140				
Heterogeneity: Tau² = 0.00; Chi² = 0.10, df = 1 (P = 0.75); I² = 0%							
Test for overall effect: Z = 4.32 (P < 0.0001)							
**1.12.5 CKD**							
Perkovic et al. 2019 (CREDENCE Trial)	110	2202	140	2199	29.9%	0.78 [0.62, 1.00]	2019
Heerspink et al. 2020 (DAPA-CKD Trial)	65	2152	80	2152	17.0%	0.81 [0.59, 1.12]	2020
Bhatt et al. 2021 (SCORED Trial)	155	5292	170	5292	38.3%	0.91 [0.74, 1.13]	2021
EMPA-KIDNEY Collaborative Group 2023 (EMPA-KIDNEY)	59	3304	69	3305	14.8%	0.86 [0.61, 1.21]	2023
**Subtotal (95% CI)**		**12950**		**12948**	**100.0%**	**0.85 [0.74, 0.97]**	
Total events	389		459				
Heterogeneity: Tau² = 0.00; Chi² = 0.90, df = 3 (P = 0.82); I² = 0%							
Test for overall effect: Z = 2.46 (P = 0.01)							
**1.12.6 Cardiac procedure**							
Raposeiras-Roubin et al. 2025 (DapaTAVI Trial)	27	605	33	617	100.0%	0.83 [0.51, 1.37]	2025
**Subtotal (95% CI)**		**605**		**617**	**100.0%**	**0.83 [0.51, 1.37]**	
Total events	27		33				
Heterogeneity: Not applicable							
Test for overall effect: Z = 0.72 (P = 0.47)							
**1.12.7 Functional MR**							
Huang et al. 2025 (DEFORM Trial)	2	52	1	52	100.0%	2.00 [0.19, 21.38]	2025
**Subtotal (95% CI)**		**52**		**52**	**100.0%**	**2.00 [0.19, 21.38]**	
Total events	2		1				
Heterogeneity: Not applicable							
Test for overall effect: Z = 0.57 (P = 0.57)							

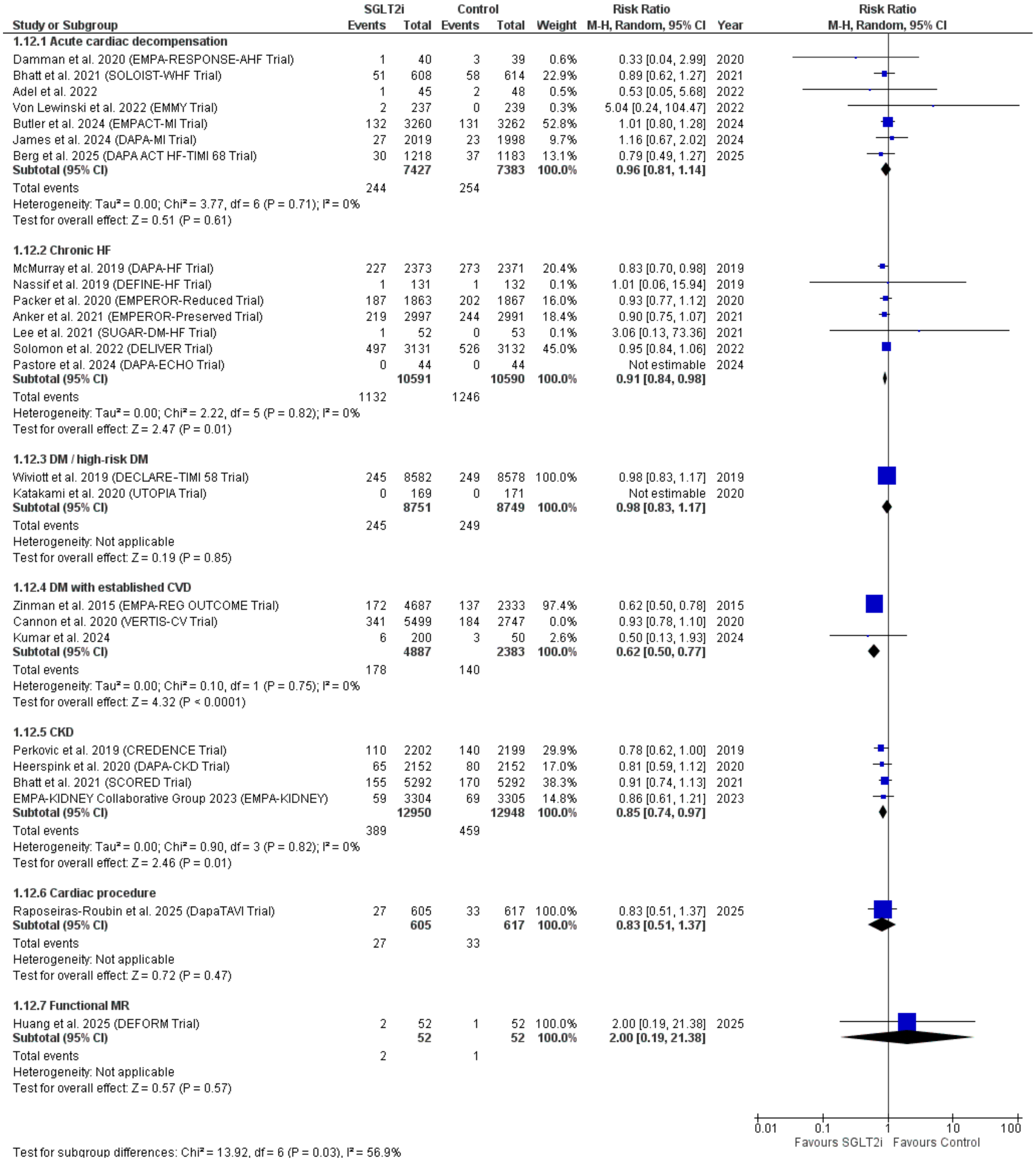

Test for subgroup differences: Chi² = 13.92, df = 6 (P = 0.03), I² = 56.9%

Figure S10. Subgroup analysis based on population for cardiovascular mortality with sensitivity analysis of the subgroup of DM with established CVD.

Study or Subgroup	SGLT2i Events	SGLT2i Total	Control Events	Control Total	Weight	Risk Ratio M-H, Random, 95% CI	Year
**1.11.1 Dapagliflozin**							
McMurray et al. 2019 (DAPA-HF Trial)	227	2373	273	2371	21.7%	0.83 [0.70, 0.98]	2019
Nassif et al. 2019 (DEFINE-HF Trial)	1	131	1	132	0.1%	1.01 [0.06, 15.94]	2019
Wiviott et al. 2019 (DECLARE-TIMI 58 Trial)	245	8582	249	8578	19.9%	0.98 [0.83, 1.17]	2019
Heerspink et al. 2020 (DAPA-CKD Trial)	65	2152	80	2152	5.8%	0.81 [0.59, 1.12]	2020
Solomon et al. 2022 (DELIVER Trial)	497	3131	526	3132	47.9%	0.95 [0.84, 1.06]	2022
James et al. 2024 (DAPA-MI Trial)	27	2019	23	1998	2.0%	1.16 [0.67, 2.02]	2024
Pastore et al. 2024 (DAPA-ECHO Trial)	0	44	0	44		Not estimable	2024
Huang et al. 2025 (DEFORM Trial)	2	52	1	52	0.1%	2.00 [0.19, 21.38]	2025
Raposeiras-Roubin et al. 2025 (DapaTAVI Trial)	27	605	33	617	2.4%	0.83 [0.51, 1.37]	2025
**Subtotal (95% CI)**		**19089**		**19076**	**100.0%**	**0.92 [0.85, 0.99]**	
Total events	1091		1186				
Heterogeneity: Tau² = 0.00; Chi² = 4.05, df = 7 (P = 0.77); I² = 0%							
Test for overall effect: Z = 2.11 (P = 0.04)							
**1.11.2 Empagliflozin**							
Zinman et al. 2015 (EMPA-REG OUTCOME Trial)	172	4687	137	2333	20.1%	0.62 [0.50, 0.78]	2015
Packer et al. 2020 (EMPEROR-Reduced Trial)	187	1863	202	1867	22.7%	0.93 [0.77, 1.12]	2020
Damman et al. 2020 (EMPA-RESPONSE-AHF Trial)	1	40	3	39	0.4%	0.33 [0.04, 2.99]	2020
Anker et al. 2021 (EMPEROR-Preserved Trial)	219	2997	244	2991	23.9%	0.90 [0.75, 1.07]	2021
Lee et al. 2021 (SUGAR-DM-HF Trial)	1	52	0	53	0.2%	3.06 [0.13, 73.36]	2021
Adel et al. 2022	1	45	2	48	0.4%	0.53 [0.05, 5.68]	2022
Von Lewinski et al. 2022 (EMMY Trial)	2	237	0	239	0.2%	5.04 [0.24, 104.47]	2022
EMPA-KIDNEY Collaborative Group 2023 (EMPA-KIDNEY)	59	3304	69	3305	12.2%	0.86 [0.61, 1.21]	2023
Kumar et al. 2024	6	200	3	50	1.2%	0.50 [0.13, 1.93]	2024
Butler et al. 2024 (EMPACT-MI Trial)	132	3260	131	3262	18.7%	1.01 [0.80, 1.28]	2024
**Subtotal (95% CI)**		**16685**		**14187**	**100.0%**	**0.85 [0.73, 0.98]**	
Total events	780		791				
Heterogeneity: Tau² = 0.02; Chi² = 14.16, df = 9 (P = 0.12); I² = 36%							
Test for overall effect: Z = 2.18 (P = 0.03)							
**1.11.3 Ertugliflozin**							
Cannon et al. 2020 (VERTIS-CV Trial)	341	5499	184	2747	100.0%	0.93 [0.78, 1.10]	2020
**Subtotal (95% CI)**		**5499**		**2747**	**100.0%**	**0.93 [0.78, 1.10]**	
Total events	341		184				
Heterogeneity: Not applicable							
Test for overall effect: Z = 0.87 (P = 0.38)							
**1.11.4 Canagliflozin**							
Perkovic et al. 2019 (CREDENCE Trial)	110	2202	140	2199	100.0%	0.78 [0.62, 1.00]	2019
**Subtotal (95% CI)**		**2202**		**2199**	**100.0%**	**0.78 [0.62, 1.00]**	
Total events	110		140				
Heterogeneity: Not applicable							
Test for overall effect: Z = 1.96 (P = 0.05)							
**1.11.5 Sotagliflozin**							
Bhatt et al. 2021 (SCORED Trial)	155	5292	170	5292	73.7%	0.91 [0.74, 1.13]	2021
Bhatt et al. 2021 (SOLOIST-WHF Trial)	51	608	58	614	26.3%	0.89 [0.62, 1.27]	2021
**Subtotal (95% CI)**		**5900**		**5906**	**100.0%**	**0.91 [0.75, 1.09]**	
Total events	206		228				
Heterogeneity: Tau² = 0.00; Chi² = 0.02, df = 1 (P = 0.90); I² = 0%							
Test for overall effect: Z = 1.06 (P = 0.29)							
**1.11.6 Tofogliflozin**							
Katakami et al. 2020 (UTOPIA Trial)	0	169	0	171		Not estimable	2020
**Subtotal (95% CI)**		**169**		**171**		**Not estimable**	
Total events	0		0				
Heterogeneity: Not applicable							
Test for overall effect: Not applicable							

Test for subgroup differences: Chi² = 2.25, df = 4 (P = 0.69), I² = 0%

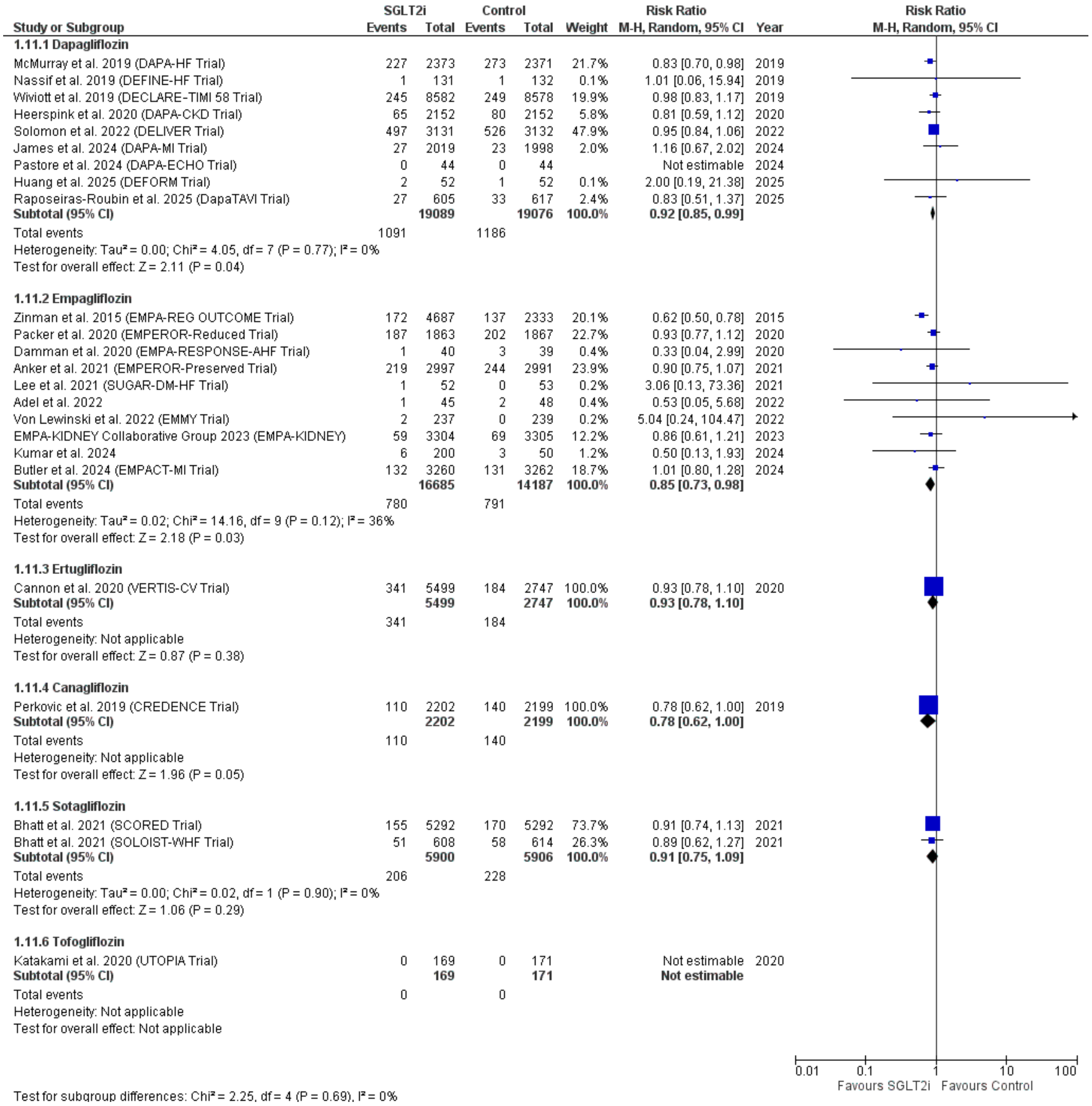

Figure S11. Subgroup analysis based on the adopted SGLT2 inhibitors.

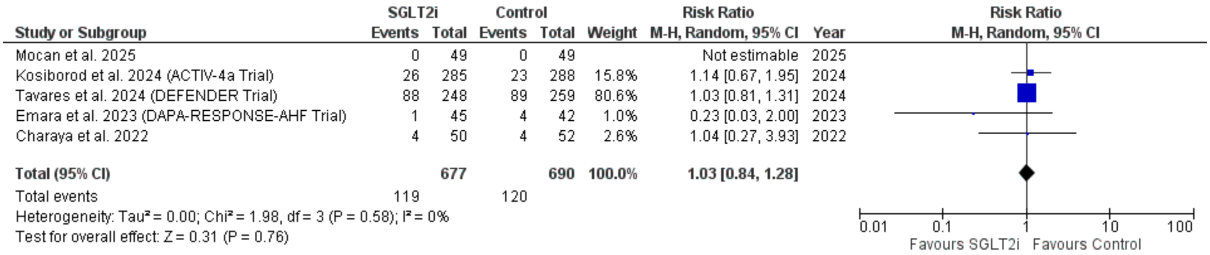

Figure S12. Forest plot of in-hospital mortality.

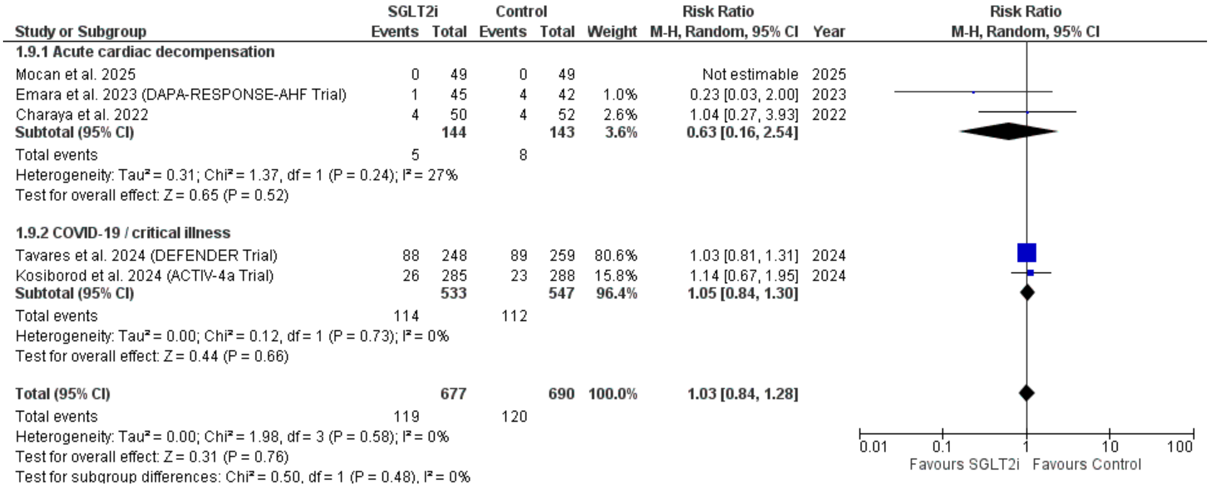

Figure S13. Subgroup analysis based on population for in-hospital mortality.

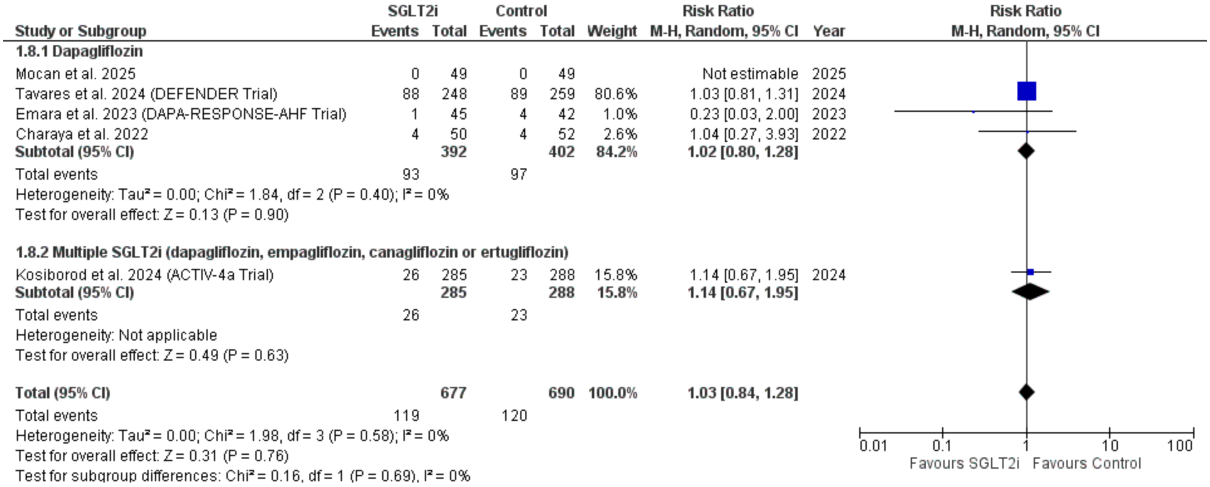

Figure S14. Subgroup analysis based on the adopted SGLT2 inhibitors.

	SGLT2i		Control			Risk Ratio	
**Study or Subgroup**	**Events**	**Total**	**Events**	**Total**	**Weight**	**M-H, Random, 95% CI**	**Year**
Cannon et al. 2020 (VERTIS-CV Trial)	0	5499	0	2747		Not estimable	2020
Heerspink et al. 2020 (DAPA-CKD Trial)	2	2152	6	2152	51.2%	0.33 [0.07, 1.65]	2020
Perkovic et al. 2019 (CREDENCE Trial)	2	2202	5	2199	48.8%	0.40 [0.08, 2.06]	2019
**Total (95% CI)**		**9853**		**7098**	**100.0%**	**0.36 [0.12, 1.14]**	
Total events	4		11				

Heterogeneity: Tau² = 0.00; Chi² = 0.02, df = 1 (P = 0.88); I² = 0%

Test for overall effect: Z = 1.73 (P = 0.08)

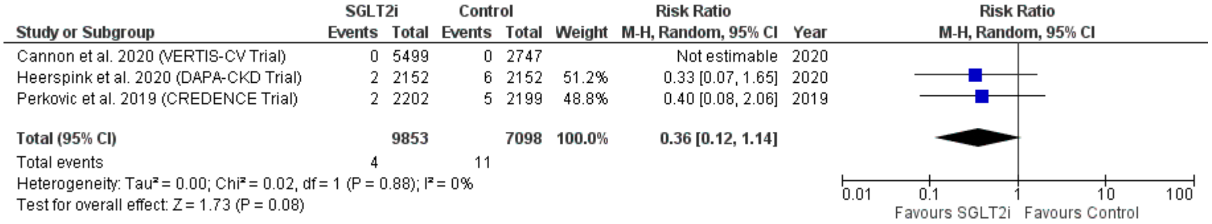

Figure S15. Forest plot of renal mortality.
